# Nuclear SPHK2/S1P induces oxidative stress and NLRP3 inflammasome activation via promoting p53 acetylation in lipopolysaccharide-induced acute lung injury

**DOI:** 10.1038/s41420-023-01320-5

**Published:** 2023-01-18

**Authors:** Linjing Gong, Yue Shen, Sijiao Wang, Xinyuan Wang, Haiying Ji, Xu Wu, Lijuan Hu, Lei Zhu

**Affiliations:** 1 Department of Respiratory and Critical Care Medicine, West China Hospital, Sichuan University, No 37 Guoxue Alley, 610041 Chengdu, Sichuan China; 2 Department of Pulmonary Medicine, Zhongshan Hospital, Fudan University, 200032 Shanghai, China; 3 Department of Orthopaedics, West China Hospital, Sichuan University, No 37 Guoxue Alley, 610041 Chengdu, Sichuan China; 4 Department of Respiratory and Critical Care Medicine, Beijing Tsinghua Changgung Hospital affiliated to Tsinghua University, 102218 Beijing, China

**Keywords:** Cell death, Respiratory distress syndrome, Acute inflammation, Experimental models of disease

## Abstract

A bulk of evidence identified that macrophages, including resident alveolar macrophages and recruited macrophages from the blood, played an important role in the pathogenesis of acute respiratory distress syndrome (ARDS). However, the molecular mechanisms of macrophages-induced acute lung injury (ALI) by facilitating oxidative stress and inflammatory responses remain unclear. Herein, we noticed that the levels of mitochondrial reactive oxygen species (mtROS), SPHK2 and activated NLRP3 inflammasome were higher in peripheral blood mononuclear cells (PBMCs) of ARDS patients than that in healthy volunteers. Similar observations were recapitulated in LPS-treated RAW264.7 and THP-1 cells. After exposure to LPS, the SPHK2 enzymatic activity, NLRP3 inflammasome activation and mtROS were significantly upregulated in macrophages. Moreover, knockdown SPHK2 via shRNA or inhibition SPHK2 could prominently decrease LPS-induced M1 macrophage polarization, oxidative stress and NLRP3 inflammasome activation. Further study indicated that upregulated SPHK2 could increase nuclear sphingosine-1-phosphate (S1P) levels and then restrict the enzyme activity of HDACs to facilitate p53 acetylation. Acetylation of p53 reinforced its binding to the specific region of the NLRP3 promoter and drove expression of NLRP3. In the in vivo experiments, it was also observed that treating with Opaganib (ABC294640), a specific SPHK2 inhibitor, could observably alleviate LPS-induced ALI, evidencing by lowered infiltration of inflammatory cells, increased M2 macrophages polarization and reduced oxidative damage in lung tissues. Besides, SPHK2 inhibition can also decrease the accumulation of acetylated p53 protein and the activation of NLRP3 inflammasome. Taken together, our results demonstrated for the first time that nuclear S1P can regulate the acetylation levels of non-histone protein through affecting HDACs enzyme activities, linking them to oxidative stress and inflammation in response to environmental signals. These data provide a theoretical basis that SPHK2 may be an effective therapeutic target of ARDS.

## Introduction

Acute respiratory distress syndrome (ARDS), with the characteristics of major hypoxemia, is a life-threatening and heterogeneous syndrome that develops in the setting of various scenarios, such as pneumonia, non-pulmonary sepsis, gastric aspiration and trauma [1]. Despite the advance of therapeutic strategies in ARDS we have made in the past few years, the morbidity and mortality rates of the syndrome still remain high among patients in the intensive-care unit (ICU) [2]. Recent years, the involvement of macrophages, including resident alveolar macrophages (AMs) and recruited macrophages from the blood, in the pathogenesis of ARDS is increasingly recognized [3, 4]. The overwhelming release of pro-inflammatory cytokines by activated macrophages leads to the accumulation of neutrophils and subsequent tissue damage. However, the role of macrophages in acute lung injury (ALI) is complex and diverse [4], and the precise molecular mechanisms of which are remains unclear.

Sphingosine kinases (SPHKs) 1 and 2, the two isoforms of SPHK with distinct subcellular localizations, have been implicated as important elements in macrophage activation to diverse stimulatory agents [5, 6]. Previous studies have demonstrated that the inhibition of SPHK1 was shown to have potential therapeutic advantage against LPS-induced lung injury [7, 8], while the properties of SPHK2 in the pathogenesis of ALI are controversial. Some researchers indicated that the population of recruited macrophages (CD11b+) suppressed AM-triggered inflammatory vascular injury via SPHK2/sphingosine-1-phosphate (S1P) signaling pathway [9]. Interestingly, a recent study revealed that SPHK2 deficiency may protect mice from pseudomonas aeruginosa (PA)-mediated lung injury via modulation of nuclear histone deacetylase1/2 (HDAC1/2) activity [10]. Besides, Syed and his colleagues documented SPHKs were directly involved in the activation of macrophage NLRP3 (the nucleotide-binding domain like receptor protein 3) inflammasome [11]. Nevertheless, the underling mechanisms of interaction between SPHKs and NLRP3 were not clear yet.

HDACs have been recognized as intracellular targets of S1P, a bioactive lipid, which is mainly generated by SPHK2 in the nucleus [12]. It has been proved that nuclear S1P can influence the dynamic turnover of histone acetylation and the target gene transcription by inhibiting enzymatic activities of HDAC1/2. p53 serves as a transcription factor that suppresses tumor development through regulation of target genes with various biological functions [13]. In addition to being a tumor suppressor protein, p53 also plays a vital role in the regulation of inflammation [14]. Previous studies presented that the stabilization and activation of protein showed correlation with the level of p53 acetylation in response to cellular stress. Moreover, acetylation of p53 was found to enhance its sequence-specific DNA-binding and promote the transcription of target genes [15, 16]. Despite substantial evidences regarding the involvement of p53 in a variety of inflammatory diseases, the exact mechanism of it in ALI is poorly understood and remains to be elucidated.

In the current study, we indicated that SPHK2 aggravated macrophages oxidative stress and NLRP3 inflammasome activation via upregulating p53 acetylation both in vitro and in vivo. Our results demonstrated for the first time that nuclear S1P can regulate the acetylation levels of non-histone protein through affecting HDACs enzyme activities, linking them to oxidative stress and inflammation in response to environmental signals. These data provide a basis that inhibition SPHK2 or knockdown *SPHK2* gene could be a therapeutic strategy for ARDS.

## Results

### The level of SphK2 protein and NLRP3 inflammasome in PBMCs of ARDS patients

**Fig. 1:**
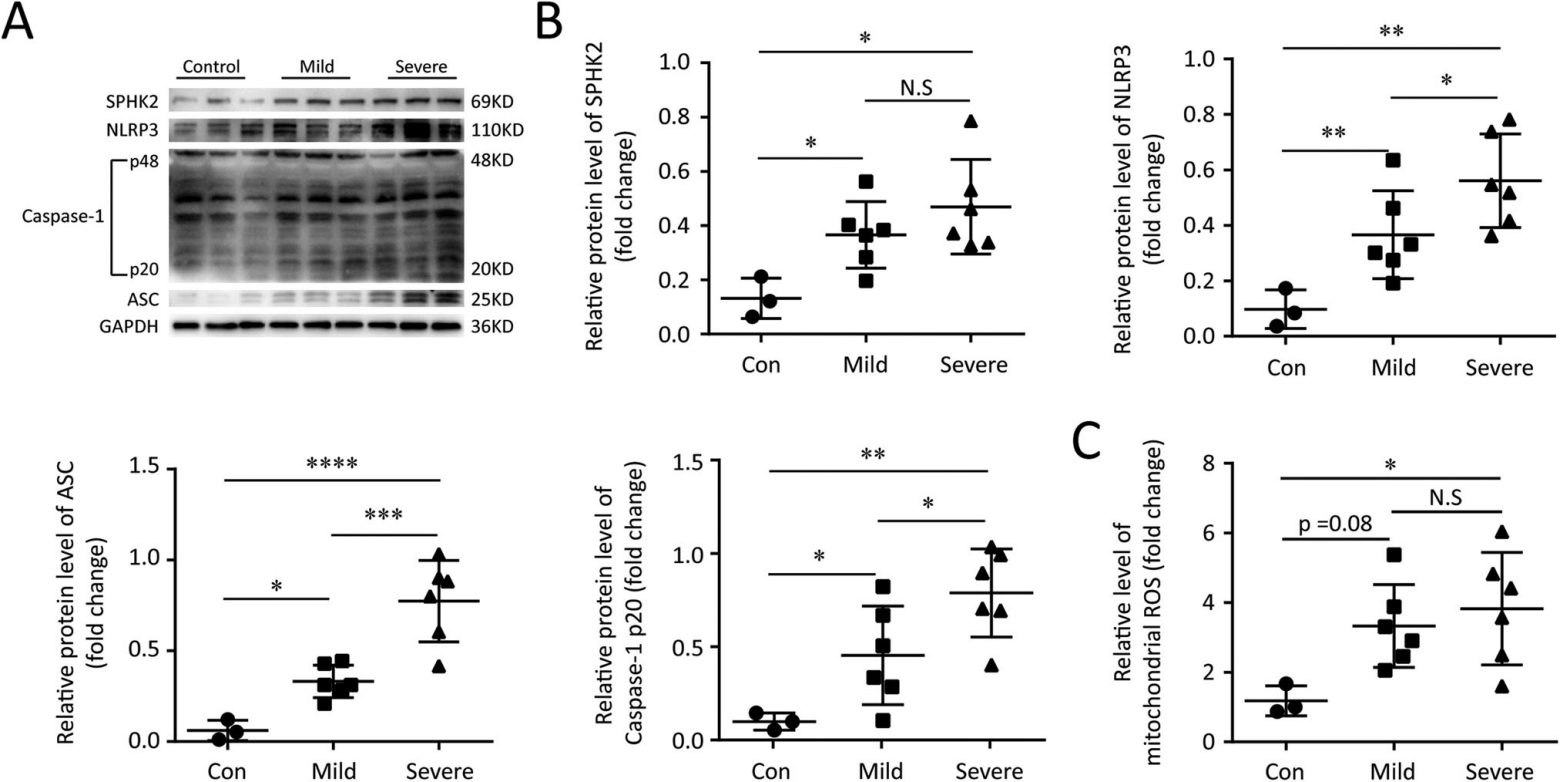
SPHK2, NLRP3 and mtROS facilitate ARDS progression. **A** Electrophoresis bands of SPHK2, NLRP3, Caspase-1, and ASC from lysates of PBMCs in different groups. GAPDH acted as an internal reference. **B** Protein levels quantified by densitometry using Image J software were shown as bar graphs. **C** mtROS levels were measured in supernatant lysates of PBMCs from subjects. Data were expressed as mean ± SEM of at least three independent experiments. *p < 0.05; **p < 0.01; ***p < 0.005; ****p < 0.001; N.S., not significant.

A great number of studies to date suggest that macrophages-triggered cytokine storm are key factors in the pathogenesis of ALI/ARDS [1]. To explore the potential relationship between the expression levels of target proteins in PBMCs and the progression in ARDS patients, we first performed immunoblotting assay. Compared with healthy volunteers, the protein levels of SPHK2, NLRP3, ASC and Caspase-1 p20 in PBMCs derived from patients with ARDS were all upregulated. Moreover, the activation of NLRP3 inflammasome in PBMCs from patients with severe ARDS was more obviously than mild ones (Fig. 1A, B and Fig. S1A). Then, the level of mtROS production was measured to detect the oxidative stress response in PBMCs. As expected, mtROS of PBMCs increased significantly in patients with severe ARDS compared to the health (Fig. 1C). These data indicated that the levels of mtROS, SPHK2 protein and activated NLRP3 inflammasome were higher in PBMCs of ARDS patients than that in the health. Furthermore, the activation degree of NLRP3 inflammasome showed positive correlation with ARDS progression.

### SPHK2 promotes LPS-induced M1 macrophage polarization, oxidative stress and NLRP3 inflammasome activation

**Fig. 2:**
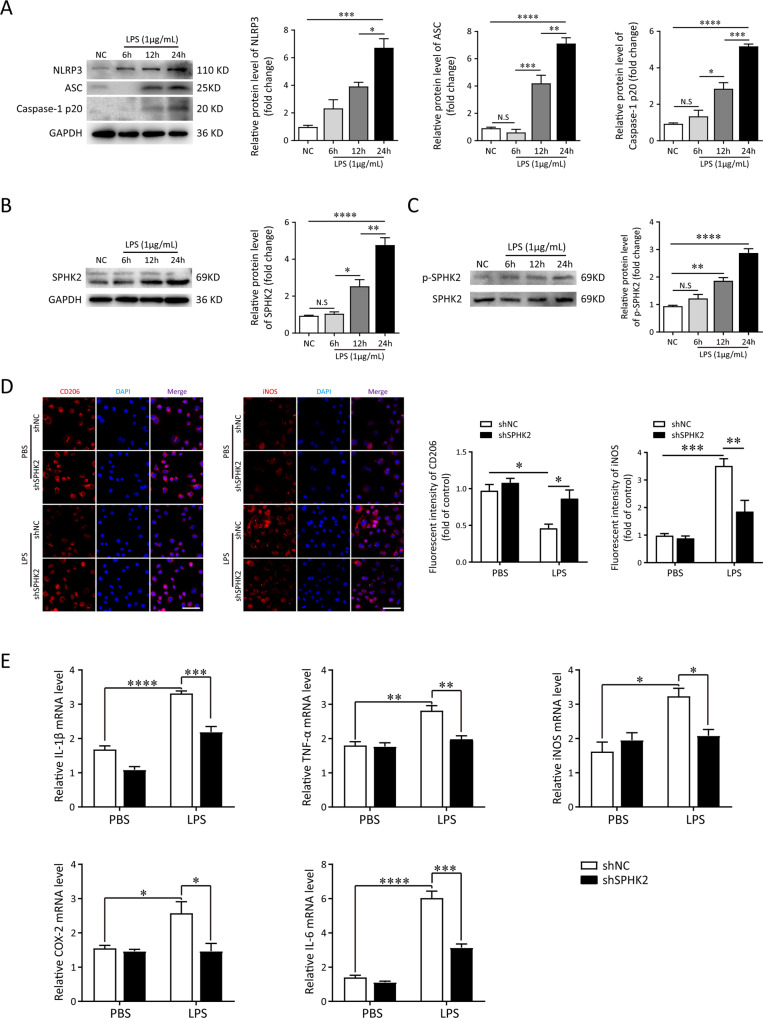
Knockdown SPHK2 restrains LPS-induced M1 macrophage polarization and inflammation. **A** RAW264.7 cells were stimulated with LPS (1 μg/mL) for 0 (NC), 6, 12, or 24 h. Western blot analysis of NLRP3, ASC and Caspase-1 p20 protein expression levels in LPS-treated RAW264.7 cells. **B** The levels of SPHK2 and **C** p-SPHK2 protein were analyzed by immunoblotting. **D** Representative immunofluorescent images of CD206 or iNOS (red) in LPS-treated RAW264.7 macrophages transfected with sh-SPHK2 or shNC. bar = 50 μm. **E** Knockdown SPHK2 could significantly decrease the production of LPS-induced inflammatory cytokines (IL-1β, TNF-α, iNOS, COX-2, and IL-6) in macrophages. Data were presented as the means ± SEM from at least three independent experiments. *p < 0.05; **p < 0.01; ***p < 0.005; ****p < 0.001; N.S., not significant.

To further determine the roles of NLRP3 inflammasome and SPHK2 in LPS-induced lung injury, the levels of NLRP3, ASC, Caspase-1 p20, SPHK2 and p-SPHK2 protein in LPS‐treated RAW264.7 cells (1 μg/mL) at 0, 6, 12, and 24 h were detected by immunoblotting or ELISA. In contrast to the control group, the activation of NLRP3 inflammasome, expression of SPHK2 and p-SPHK2 in macrophages were elicited by LPS exposure. The elevated levels of activated NLRP3 inflammasome and SPHK2 enzyme activity were positively correlated with the stimulation of LPS in a time‐dependent manner (Fig. 2A, B; Figs. S1B and S2). These data indicated that SPHK2 and NLRP3 inflammasome activation might be essential for the development of ALI. Next, RAW264.7 cells were transfected with shRNA to knockdown the expression of SPHK2. As shown in Fig. 2C, SPHK2 shRNA3 showed optimal results, and hence, was selected for the subsequent experiments. According to the results of immunofluorescent staining, the downregulation of SPHK2 significantly increased the number of RAW264.7 cells positive for CD206 (M2 marker) and decreased the number of macrophages positive for iNOS (M1 marker) with LPS stimulation (Fig. 2D). Moreover, the results of qRT-PCR showed that knockdown SPHK2 obviously inhibited the production of inflammatory cytokines (such as IL-1β, TNF-α, iNOS, COX-2 and IL-6) in LPS-induced macrophages (Fig. 2E). These results showed that SPHK2 could promote LPS-induced M1 macrophage polarization and subsequent inflammation in vitro.

**Fig. 3:**
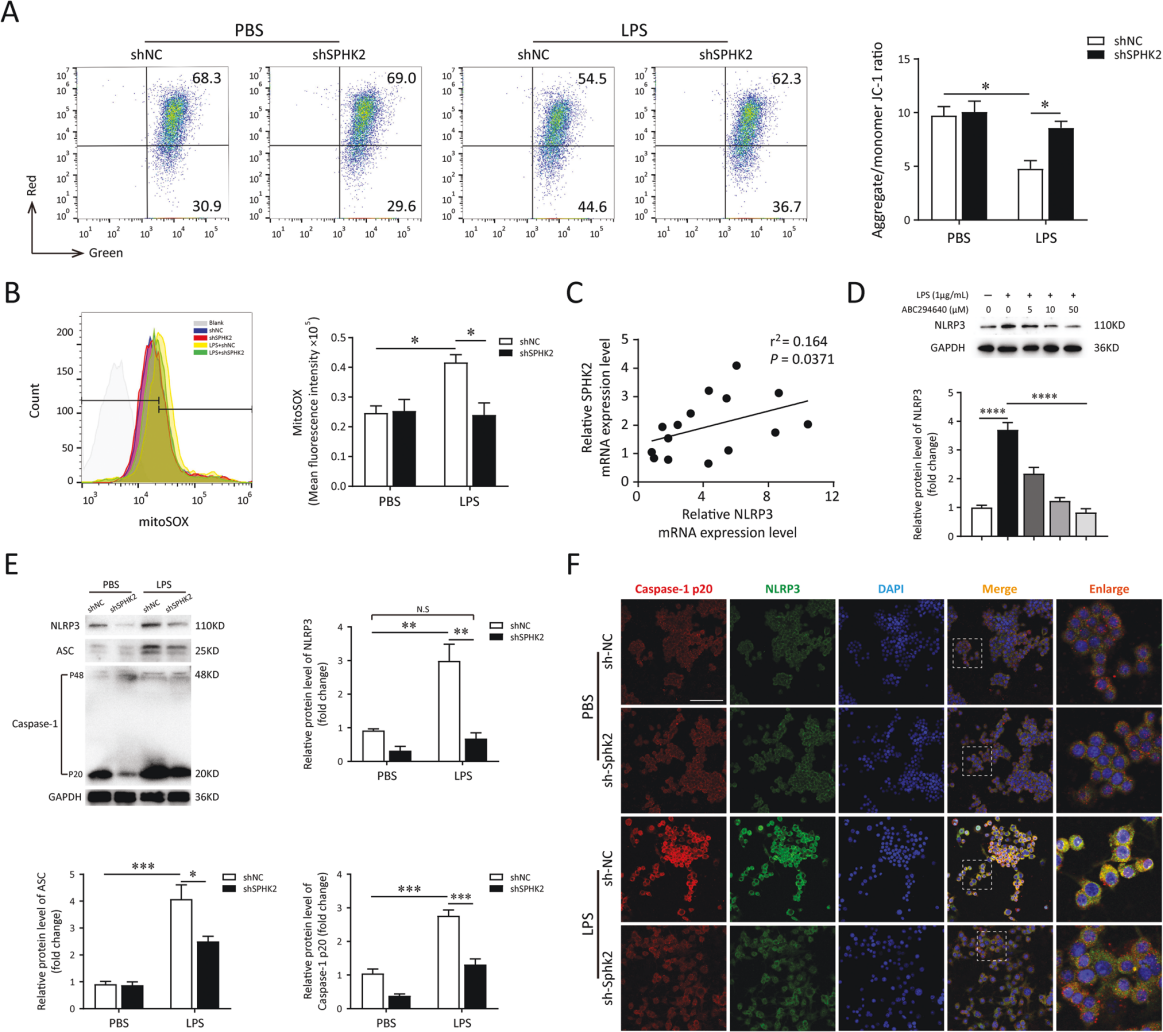
SPHK2 is essential for LPS-triggered oxidative stress and NLRP3 inflammasome activation in macrophage. **A** sh-SPHK2 or shNC transfected RAW264.7 cells were stained with JC-1 and analyzed quantitation of fluorescence intensity by flow cytometry. **B** sh-SPHK2 or shNC transfected RAW264.7 cells were stained with MitoSOX and analyzed by flow cytometry. LPS-elicited mtROS generation was inhibited by SPHK2 knockdown. **C** qRT-PCR analysis was performed to determine the correlation between SPHK2 and NLRP3 mRNA levels for individual sample. **D** Western blots of NLRP3 in RAW264.7 cells exposed to PBS or LPS, treating with or without ABC294640. **E** Cell lysates were immunoblotted for NLRP3, ASC, and Caspase-1 p20 proteins in RAW264.7 cells. **F** Representative confocal microscopic images of gene-modified RAW264.7 cells co-localization with Caspase-1 p20 (red) and NLRP3 (green). bar = 100 μm. Data were presented as the means ± SEM from at least three independent experiments. *p < 0.05; **p < 0.01; ***p < 0.005; N.S., not significant.

Mitochondrial dysfunction is a hallmark of oxidative stress, so we assessed mitochondrial function in LPS-treated macrophages via detecting the levels of MMP and mtROS. We found that SPHK2 knockdown markedly restored the disrupted MMP (*p* < 0.05, 0.99 ± 0.07% vs. 1.83 ± 0.06%) and attenuated the release of mtROS (*p* < 0.05, 58.2 ± 0.87% vs. 40.5 ± 1.59%) in macrophages exposed to LPS (Fig. 3A, B). Then, to investigate whether SPHK2 could modulate the activation of NLRP3 inflammasome, we analyzed the correlation between SPHK2 and NLRP3 expression level following the LPS exposure. As illustrated in Fig. 3C, results of qRT-PCR revealed the positive correlation between the mRNA expressions of SPHK2 and NLRP3. Western blot analysis also showed a positive correlation between SPHK2 activity and NLRP3 protein expression (Fig. 3D). Besides, both immunoblot and immunofluorescence images demonstrated that the accumulation of NLRP3, ASC and cleaved caspase-1 (also detected through ELISA; Fig. S1C) induced by LPS was substantially abolished by downregulation of SPHK2 (Fig. 3E, F). Similar phenomena have also been demonstrated in human-derived monocyte cell line THP-1 via using ABC294640 (Fig. S3A–C). Taken together, these findings suggested that SPHK2 played a positive role in LPS-triggered oxidative stress and NLRP3 inflammasome activation in macrophage.

### Nuclear SPHK2/S1P signaling facilitates LPS-evoked NLRP3 inflammasome activation in macrophage via modulating p53 acetylation

**Fig. 4:**
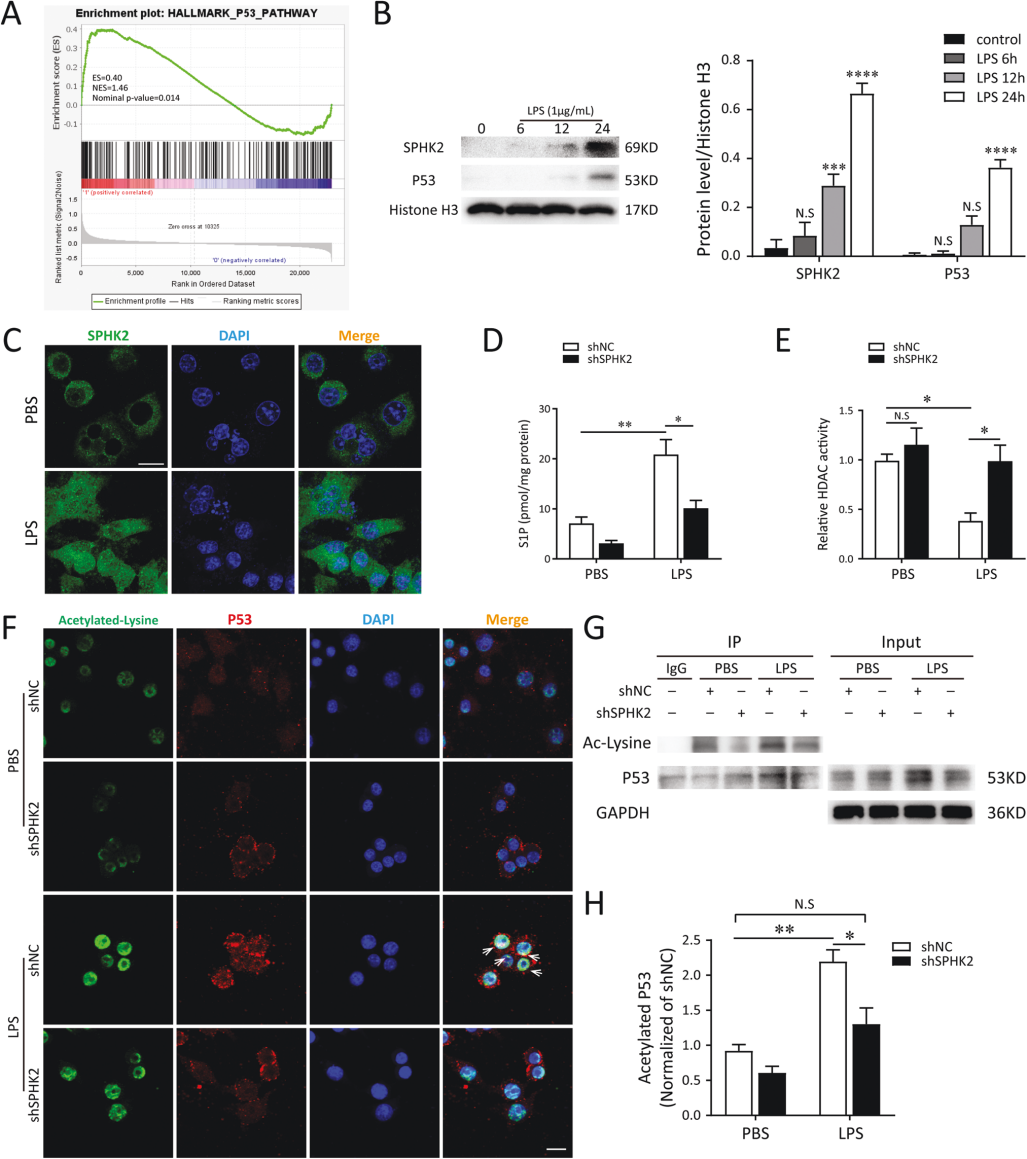
The activation of nuclear SPHK2/S1P signaling promotes p53 acetylation in LPS-induced macrophages. **A** Gene set enrichment analysis (GSEA) analysis identified the enriched gene sets in AMs exposed to saline or LPS from the same individuals (*n* = 7). Exposure to LPS was associated with p53 pathway. NES, normalized enrichment score. **B** Expressions of nuclear SPHK2 and p53 protein were detected by western blot after LPS stimulation. **C** Representative confocal microscopic images of SPHK2 (green) in RAW264.7 cells with or without LPS stimulations. bar = 50 μm. Gene-modified RAW264.7 macrophages were treated with 1 μg/mL LPS for 24 h. S1P levels (**D**) in the nuclear fraction was determined by LC-MS/MS and cell lysates were detected for HDACs activity (**E**) using a commercial kit. **F** Representative confocal microscopic images of gene-modified RAW264.7 cells co-localization with Acetylated-lysine (green) and p53 (red). bar = 25 μm. **G** and **H** Acetylation of p53 after 1 μg/mL LPS exposure was determined by Co-IP with an anti-p53 antibody, followed by western blot analysis of acetylated-lysine. Data were presented as the means ± SEM from at least 3 independent experiments. *p < 0.05; **p < 0.01; ***p < 0.005; ****p < 0.001; N.S., not significant.

GSEA analysis from the GSE4088 dataset (https://www.ncbi.nlm.nih.gov/geo/query/acc.cgi?acc=GSE4088) identified AMs exposed to LPS in vivo potentially associated with the enhanced p53 pathway (Fig. 4A). In order to clarify whether p53 is involved in SPHK2-mediated NLRP3 inflammasome activation, the expressions of SPHK2 and p53 in the nuclear fraction of LPS-stimulated macrophage were first detected. Compared to the control group, LPS significantly promoted the protein expressions of SPHK2 and p53 in macrophage nucleus (Fig. 4B, C). Acetylation of p53 is an important reversible enzymatic process that occurs in response to oxidative stress and DNA damage, and is indispensable for p53 transcriptional activity [17]. To understand the role of p53 in SPHK2-mediated inflammation, we then investigate if p53 transcriptional activity is regulated by the nuclear SPHK2/S1P signaling pathway. After exposing to LPS, the level of S1P was substantially increased compared with the control group, while the enzymatic activity of HDACs was correspondingly decreased. SPHK2 knockdown obviously reduced S1P level and restored HDACs enzymatic activity in nucleus of LPS-treated macrophages (Fig. 4D, E). Double immunofluorescent staining for acetylated-lysine (green) and p53 (red) indicated that the co-localization of p53 and acetylated-lysine reinforced by LPS treatment was dramatically counteracted in SPHK2-deficient cells (Fig. 4F). Further co-IP assay and western blotting confirmed that LPS exposure could enhance the p53 acetylation. Notably, the increase of p53 acetylation induced by LPS was neutralized by the downregulation or inhibition of SPHK2 (Fig. 4G, H and Fig. S3D). These data demonstrated that nuclear S1P promoted p53 acetylation via inhibiting the enzymatic activity of HDACs in LPS-treated macrophages.

**Fig. 5:**
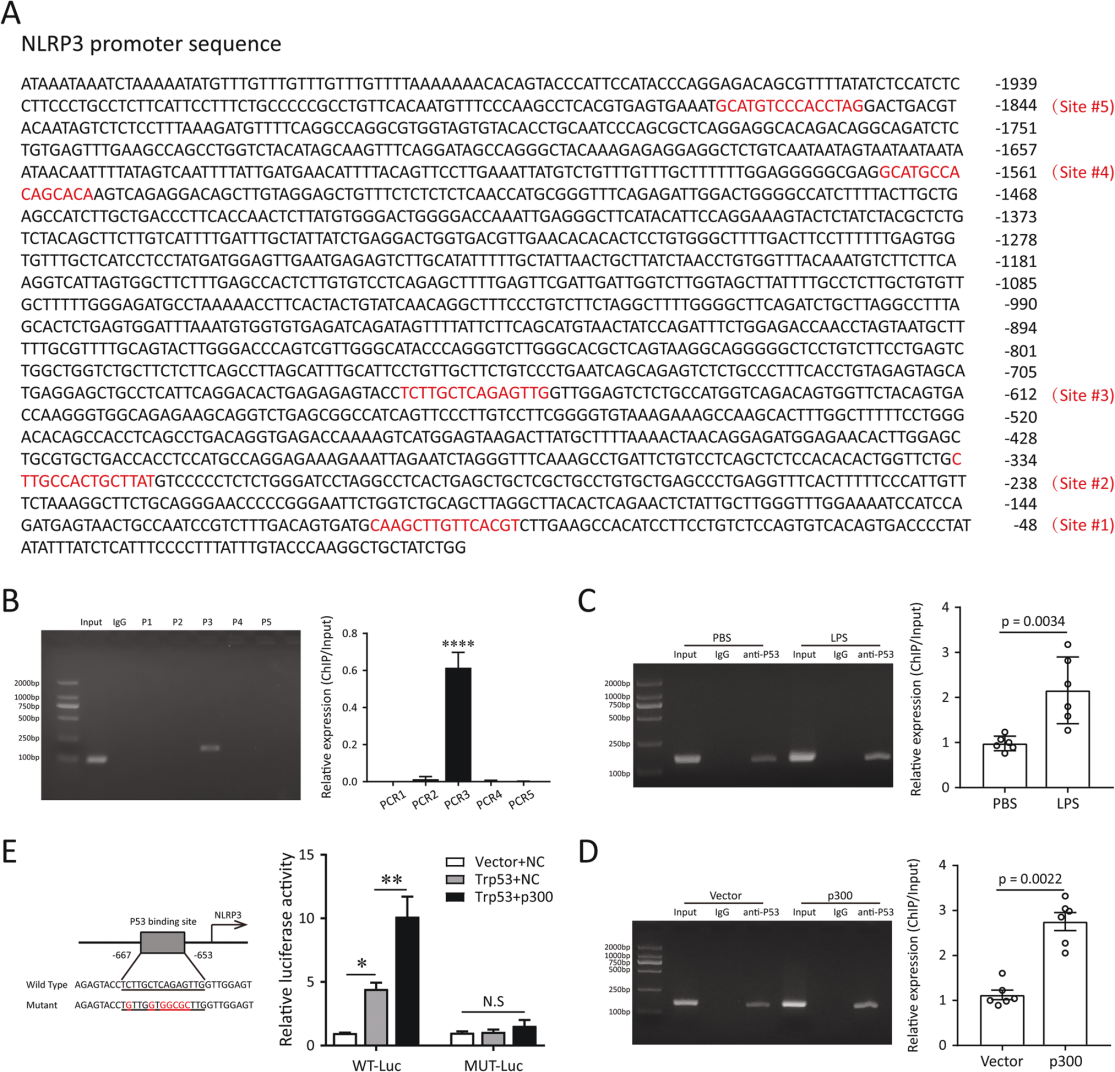
p53 acetylation upregulates the transcription of NLRP3. **A** The sequences from position −2035 to 0 bp of NLRP3 promoter. The predicted potential binding sites of p53 are highlighted in red. **B** ChIP-PCR was performed with p53 antibody in RAW264.7 cells to explore the enrichment of potential binding sequences of NLRP3 promoter region. The qRT-PCR products were validated by agarose gel electrophoresis. **C** ChIP analysis of RAW264.7 cells pretreated with or without LPS (1 μg/mL) for 24 h, as well as (**D**) RAW264.7 cells transfected with or without p300 plasmids for 48 h. Chromatin was immunoprecipitated with anti-p53 antibodies and then subjected to PCR and agarose gel electrophoresis analysis, respectively. **E** Effects of p53 acetylation on NLRP3 expression detected using dual-luciferase reporter assay. Data were presented as the means ± SEM from at least three independent experiments. *p < 0.05; **p < 0.01; ****p < 0.001; N.S., not significant.

Since p53 is a well-known transcription factor, we wonder if p53 could bind to NLRP3 promoter and regulate its transcription. We subsequently conducted bioinformatics analysis to predict 5 potential promoter binding sites of NLRP3 to p53 via JASPAR (Fig. 5A, http://jaspar.genereg.net/), and designed five pairs of specific primers (Table S2). Results of ChIP assays indicated that the regulatory region between −667 and −653 bp was responsible for p53-mediated NLRP3 promoter regulation, whereas the others failed to bind with p53 (Fig. 5B). Both LPS exposure and p300 overexpression could increase the recruitment of p53 to this region of the promoter of *NLRP3* gene in RAW264.7 cells (Fig. 5C, D). A luciferase reporter assay also demonstrated that co-transfection of the p53 expression plasmid with the WT-NLRP3 promoter luciferase reporter exhibited nearly fourfold increased luciferase activity compared with mutant one. In addition, the luciferase activity of WT reporter could be further activated with p300 overexpression (Fig. 5E). Together, these results supported our hypothesis that p53 bound to the putative region of the NLRP3 promoter and facilitated NLRP3 transcription. Furthermore, LPS stimulation could enhance this process by promoting p53 acetylation in macrophages.

### Inhibition of SPHK2 ameliorates LPS-induced lung oxidative injury and inflammation

**Fig. 6:**
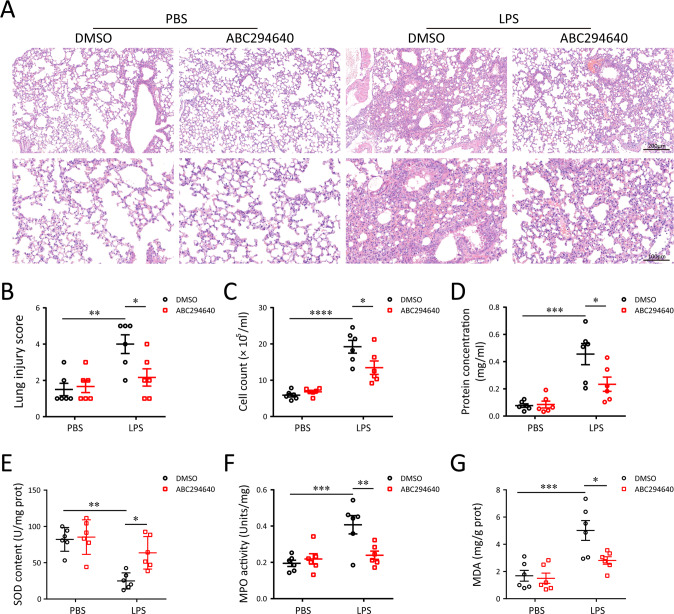
Inhibition of SPHK2 with ABC294640 protects the lung against LPS-driven oxidative injury in mice (*n* = 6). **A** Representative lung sections from PBS group, ABC294640 group, LPS group and LPS + ABC294640 group stained by H&E. **B** Lung injury score, **C** inflammatory cell count and **D** BALF protein concentration among different groups. **E** SOD content, **F** MPO activities, and **G** MDA levels were measure in lung tissue homogenates. Data were presented as the means ± SEM. *p < 0.05; **p < 0.01; ***p < 0.005; ****p < 0.001.

To determine whether blocking of SPHK2 activity could ameliorate LPS-induced pulmonary injury in vivo, we performed histological examination on lung tissues. When compared with vehicle -challenged mice, treatment with ABC294640 6 h post-infection evidently attenuated LPS-induced lung injury, reflected by less hemorrhage, and decreased thickness of the alveolar wall (Fig. 6A). The lung injury scores of each group were quantitated simultaneously. Consistent with histological examinations, the score tended to be reduced by ABC294640 treatment in comparison with that of the LPS group (Fig. 6B). Furthermore, LPS-triggered tissue injury, infiltration of inflammatory cells, as well as the concentrations of protein in BALF were all significantly reduced in mice treated with ABC294640 compared with vehicle challenged ones (Fig. 6C, D). It is important to emphasize that LPS-induced lung injury to be associated with oxidative stress. Hence, to examine the levels of oxidative stress in lung tissues after LPS exposure, the content of SOD, MPO and MDA was measured. We found that exposed mice to LPS were shown to have the decreased SOD contents, and increased MPO activities and MDA levels. Nonetheless, compared with the LPS group, treatment with ABC294640 ameliorated the oxidative damage in lung tissues (Fig. 6E–G).

**Fig. 7:**
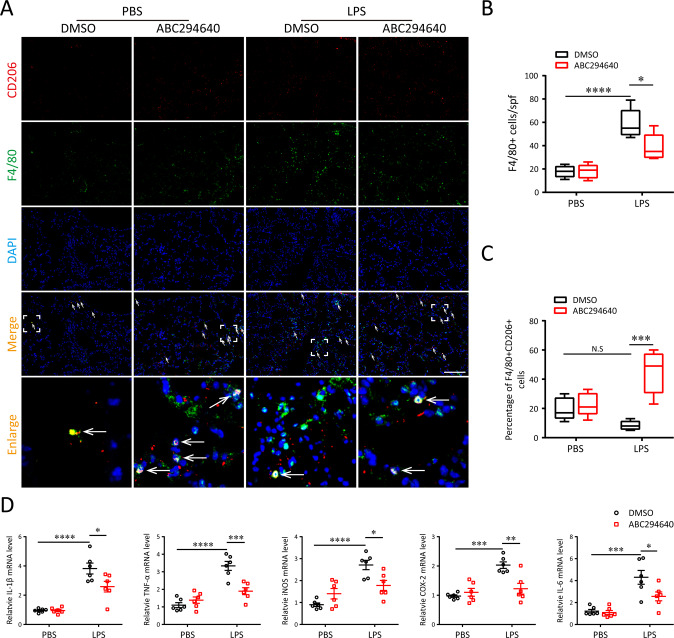
Inhibition of SPHK2 with ABC294640 ameliorates LPS-induced inflammatory lung injury in mice (*n* = 6). **A** Representative images of double-labeled with CD206 and F4/80 (M2 macrophage, white arrow) in lung revealed the increased (**B**) macrophages infiltration and decreased (**C**) M2 macrophage polarization after 5 mg/kg LPS exposure, which changes could be weakened with the inhibition of SPHK2. **D** mRNA levels for various inflammatory cytokine genes (TNF-α, iNOS, COX-2, IL-6, and IL-1β) among different groups. Data were presented as the means ± SEM. *p < 0.05; **p < 0.01; ***p < 0.005; ****p < 0.001; N.S, not significant.

Since macrophages were regarded as a predominant contributor to the pathogenesis of ALI, we wonder whether inhibition of SPHK2 activity influences the behavioral changes of macrophages during LPS exposure in vivo. As illustrated in Fig. 7A–C, treatment with ABC294640 could reduce the infiltration of F4/80^+^ macrophages into lung tissue after exposure to LPS. Moreover, blocking of SPHK2 activity obviously induced M2 macrophage polarization. We then tested the effects of ABC294640 on the levels of inflammatory mediators in lung tissue with qRT-PCR. The results showed that ABC294640 treatment visibly suppressed the accumulation of LPS-induced inflammatory cytokines (Fig. 7D). According to the above results, our data revealed that inhibition of SPHK2 could alleviate macrophage-evoked oxidative injury and inflammatory reaction in lung tissues.

### Inhibition of SPHK2 suppresses p53 acetylation and NLRP3 inflammasome activation in vivo

**Fig. 8:**
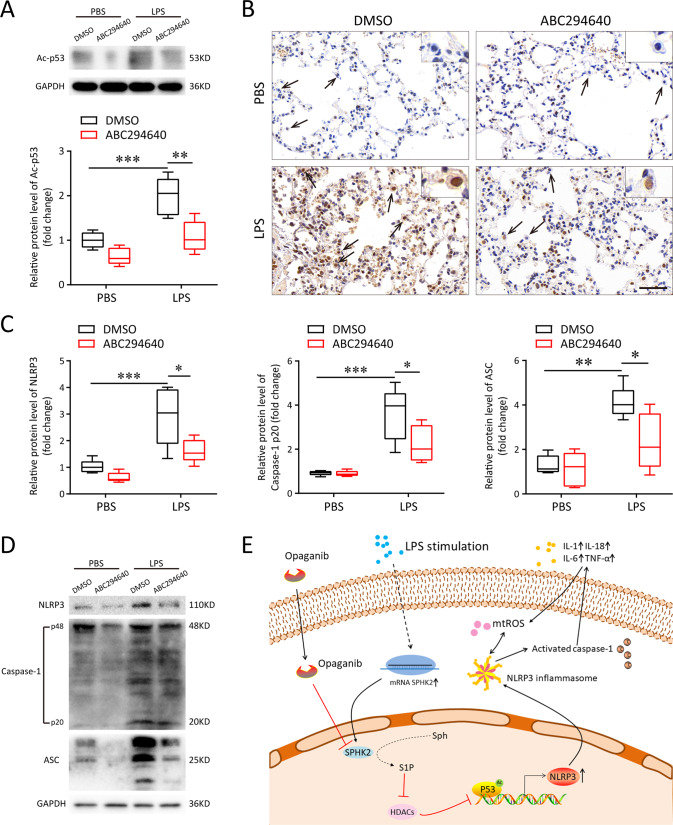
Inhibition of SPHK2 with ABC294640 suppresses the acetylation levels of p53 as well as the activation of NLRP3 inflammasome in vivo. **A** Western blots of acetyl-p53 in the lung tissues from mice exposed to PBS or LPS, treating with DMSO or ABC294640. **B** Representative images of immunostaining for NLRP3 in the lung were shown (alveolar macrophages, black arrow). bar = 50 μm. **C** and **D** Western blots of NLRP3, Caspase-1 p20 and ASC in the lung tissues from mice. **E** The proposed model to illustrate the underlying mechanism that SPHK2/NLRP3 axis is implicated in the progression of ARDS. Data were presented as the means ± SEM from at least 3 independent experiments. *p < 0.05; **p < 0.01; ***p < 0.005.

Next, the levels of p53 acetylation and NLRP3 inflammasome activation were also detected in lung tissues via immunoblotting and immunohistochemical staining. In line with the in vitro results, our in vivo data indicated that LPS alone remarkably increased the protein levels of acetylated p53. However, treatment with ABC294640 could decrease p53 acetylation (Fig. 8A). As displayed in Fig. 8B, NLRP3 was highly expressed in AMs of mice exposed to LPS. In contrast, the immunohistochemical staining showed the NLRP3 was faintly stained in the AMs of mice exposed to LPS that treatment with ABC294640. Western blotting was applied to the lung tissue samples to further detect the activation of NLRP3 inflammasome. The levels of NLRP3, ASC and activated caspase-1 in lung tissues of LPS-challenged mice were all upregulated compared to the PBS group. Furthermore, administration of ABC294640 significantly decreased the expression levels of NLRP3, ASC and cleaved caspase-1 (Fig. 8C, D). In summary, our data indicated that knockdown or inhibition of SPHK2 could partially suppress the LPS-induced NLRP3 inflammasome activation and subsequent inflammatory reaction in macrophages through modulating acetylation levels of p53 both in vitro and in vivo (Fig. 8E).

## Discussions

At present, the molecular mechanisms of macrophages leading to ALI by facilitating oxidative stress and inflammatory response are not fully understood [18]. Our present study indicated that the levels of SPHK2 and NLRP3 inflammasome in PBMCs were positively correlated with the occurrence and development of ARDS. Macrophages are a robust source of oxidative stress, production of which are critical for self-activation and the accumulation of pro-inflammatory cytokines. In our in vitro experiments, SPHK2 expression was increased significantly by LPS, and then promoted the activation of NLRP3 inflammasome. Further study showed that upregulated SPHK2 could increase the levels of nuclear S1P, that restricted the enzyme activity of HDACs. Whereafter, increased levels of p53 acetylation further accelerated NLRP3 transcription and triggered an inflammatory cascade. Moreover, we also indicated that SPHK2/p53/NLRP3 axis was implicated in LPS-induced lung injury in vivo. Blocking of SPHK2 through its inhibitor ABC294640 could effectively alleviate tissues oxidative damage that induction with LPS. Our study reveals a novel role of SPHK2 in macrophage-mediated lung injury and suggests SPHK2 as a potential therapeutic target of ARDS. However, it should be noted that previous studies have also suggested neutrophils-mediated inflammatory responses play an important role in the pathogenesis of several respiratory diseases [19], among which S1P signaling and NLRP3 inflammasome might be vital ones [20, 21]. In this study, we mainly investigated the role of SPHK2-NLRP3 signaling pathway in macrophage-triggered inflammatory storm, whether there is a similar mechanism in neutrophils remains unclear and is worthy of further study in the following experiments.

Many studies have shown that macrophages are key regulators in the pathogenesis of ALI/ARDS, including COVID-19 [22, 23]. In acute exudate stage of ARDS, the sustained M1 polarization of AMs can release a large number of pro-inflammatory cytokines, including TNF-α, IL-1β, ROS, and then cause acute inflammatory injury [24]. Therefore, regulation of macrophage polarization may be a therapeutic strategy to alleviate ALI. Cui and his colleagues found that conditional knockout of macrophage lncRNA Malat1 could mitigate LPS-induced systemic and pulmonary inflammation by restraining M1-type macrophage activation [25]. Recently, researchers have noticed a bioactive nanomaterial that can specifically induce M2 macrophage polarization both in vivo and in vitro to reduce lung inflammation and injury, which was expected to become a new generation of treatment for ALI [26]. However, it was also proposed that M1 AMs could actually lower the expression of inflammatory factors by producing high level of amphiregulin, thus exhibiting a protective effect on lung tissue [27]. In short, the pathological changes of ALI/ARDS are a continuous process, and the effects of different degrees of inflammation in different periods may be opposite. Thus, it might be a research direction of ARDS treatment in the future to give precise interventions in a specific period [28].

Currently, some studies pointed out that sphingolipids and its metabolites played an important role in normal cell functions and in the pathogenesis of human pulmonary disorders, such as ARDS [8, 9]. S1P is a bioactive lipid that is generated by the action of two sphingosine kinases (SPHK1 and SPHK2); among them, SPHK2 is mainly distributed in the nuclear, endoplasmic reticulum and mitochondria [29]. The roles of these two kinases during the onset of ALI are all quite complex. Some studies have reported that neither of the two lipid kinases played an important role in signaling cascades in inflammation [30]. However, other studies noted that SPHK2 could suppress mtROS, limit cytokines release from human inflammatory macrophages [6], and exhibit anti-inflammatory properties in recruited CD11b + macrophages [31]. Previous finding has also indicated that genetic deletion of SPHK2, but not SPHK1, in mice conferred protection from PA-mediated lung inflammation [10]. Interestingly, several reports suggested that SPHK1 inhibition showed a clear potential therapeutic advantage against sepsis [8]. These phenomena may be related to different stimulus conditions, as well as the protein subcellular localization. In the present study, we discovered that SPHK2 was dramatically increased in PBMCs of ARDS patients and LPS-stimulated macrophages; overexpressed SPHK2 was positively associated with oxidative stress and inflammatory cascade in macrophages. Similar to our findings, other researches also showed that intracellular S1P signaling system participates in the regulation of macrophage function and inflammatory response [32]. Additionally, SPHK2 was identified as a critical regulator of inflammatory injury; reducing SPHK2 activity by genetic or pharmacological manipulation could in large part promote macrophage polarization to the M2 phenotype [33].

It has been proven that nuclear SPHK2/S1P signaling can regulate inflammation via influencing the enzymatic activity of HDACs and the transcription of target genes [10, 12, 34]. p53 is best known as a transcription factor [35], and it is the first non-histone protein shown to be acetylated by histone acetyltransferases (HATs) [17]. A number of more recent in vivo models have underscored the importance of this type of modification for p53 activity [17]. During stress responses, acetylation of p53 reinforced its function of a nuclear transcription factor to drive expression of relevant stress-response genes. Deletion of HDAC1/2 has been shown to maintain significantly high levels of p53 acetylation at lysine 370, 379 and 383, and this post-translational modification improved the stability of p53 and transcriptional activity of downstream target genes [36]. In addition, it has also been documented that dysacetylation of p53 caused by SIRT1 deficiency can promote inflammatory response and endothelial dysfunction [37]. In our work, we firstly predicted the potential binding site of p53 as a transcription factor to NLRP3 promoter through the JASPAR database. Then, co-IP assay was performed to prove that the acetylation of p53 is regulated by nuclear SPHK2/S1P signal. Finally, ChIP and double-luciferase reporter assays verified that the increased acetylation of p53 reinforced the activation of NLRP3 promoter transfer activity. Based on these findings, our study is the first to identify nuclear S1P could influence the delicate balance and dynamic turnover of p53 acetylation and the transcription of target genes.

Opaganib (ABC294640) is a specific SPHK2 inhibitor that prevents sphingosine from being phosphorylated into its active form by competitively binding SPHK2, thereby effectively reducing intracellular S1P levels and confining inflammatory signaling pathways [38]. Opaganib has been shown to reduce mortality in mouse models of influenza virus [39] and improve lung injury induced by Pseudomonas aeruginosa (both pre-infection and post-infection) [10]. Furthermore, in a completed clinical trial of Compassionate use to treat patients with severe COVID-19 in a small cohort (ClinicalTrials.gov Identifie: NCT04435106), Opaganib was shown to be safe and well tolerated. There were substantial improvements in clinical outcomes and markers of inflammation in patients treated with the drug, suggesting that Opaganib was a safe and potent candidate for treatment of ARDS patients. Consistent with the above experimental results, our in vivo data showed that Opaganib significantly reduced LPS-induced ALI, evidencing by lowered infiltration of neutrophils and F4/80^+^ macrophages in lung tissues, increased M2 macrophages polarization, reduced concentration of BALF protein and decreased MPO activity. Mechanically, Opaganib can also prevent NLRP3 inflammasome activation via inhibition of SPHK2 and p53 acetylation in vivo. Although it may have potential therapeutic implications, many targeted antibodies and small-molecule inhibitors are also associated with organ impairment because of off-target toxicity [40]. How to reduce the off-target effect or mitigate the toxicity after the off-target is the difficulty and hotspot of current research. At present, there are few studies on the off-target effect of Opaganib, which may be a focus of attention in the future. Anyhow, the exact clinical efficacy of Opaganib in the treatment of ARDS is still nor clear and warrant further research.

## Conclusions

In conclusion, blocking of SPHK2 significantly reverses the LPS-induced oxidative injury and inflammation in lung tissues of mice. In particular, our observations show that inhibition of SPHK2 prevents LPS-induced M1 macrophage polarization and subsequent inflammatory cascade by restricting oxidative stress and NLRP3 inflammasome activation via inhibiting acetylation of p53. Taken together, our preliminary experiments suggest that SPHK2 and mtROS-NLRP3 signal play key roles in the pathogenic processes driven by M1 macrophages in ARDS patients, which can be offset by Opaganib-mediated SPHK2 inhibition. Overall, these findings demonstrate that SPHK2 may be a potential therapeutic target for ARDS.

## Material and methods

### Animals, cell lines, and reagents

The C57BL/6J mice (6–8 weeks old, 18–22 g, *n* = 24) were purchased from the Model Animal Research Center of Nanjing University and raised in specific pathogen-free (SPF) conditions. The murine-derived macrophage cell line RAW264.7 and human-derived monocyte cell line THP-1 were purchased from Shanghai Zhong Qiao Xin Zhou Biotechnology Co., Ltd. Lipopolysaccharide (LPS, Escherichia coli derived) was obtained from Sigma Chemical (USA) and Opaganib (ABC294640) was obtained from AbMole. We obtained MitoSOX (M36008) from ThermoFisher scientific and mitochondrial membrane potential assay kit with JC-1 (C2006) from Beyitome. Cleaved caspase-1 p20 ELISA Kit was purchased from R&D Systems (USA) or IBL International (Germany), Inc. (USA). SPHK2 (ab264042), NLRP3 (ab214185, ab4207 for immunofluorescence), and acetyl-p53 (ab183544) were obtained from Abcam; ASC (apoptosis-associated speck-like protein, #67824), p53 (#2524), Acetylated-lysine (#9441), Histone H3 (#4499), CD206 (#24595) and iNOS (#13120) were obtained from Cell Signal Technology (CST); Caspase-1 p20 (sc-398715) was obtained from Santa Cruz Biotechnology; phospho-SPHK2 (p-SPHK2, #AF3532) was obtained from Affinity Biosciences; GAPDH (AF1186) was obtained from Beyitome. Fluorescence secondary antibodies were purchased from Jackson ImmunoResearch. SPHK2 small hairpin RNA (shRNA) plasmid was purchased from GenePharma (Shanghai, China). The sequence of sh-SPHK2 was as follows: 5’-GCCAATGATCTCTGAAGCTGG-3’.

### Patient samples

All subjects were recruited from the respiratory intensive-care unit (RICU) of Zhongshan Hospital, Fudan University, China, with approval by the Ethics Committee of the Zhongshan Hospital, Fudan University. Written informed consent was obtained from every participant. To investigate the relationship between the expression levels of target genes and the progression in ARDS patients, we recruited 6 participants with mild ARDS, 6 participants with moderate/severe ARDS, and 3 healthy control participants. The inclusion criteria and severity classification of ARDS referred to the Berlin definition proposed in 2012 [41]. Approximately 15 mL of peripheral venous blood sample was collected from each participant within 24 h of admission using heparin (50 U/mL) as an anticoagulant. Peripheral blood mononuclear cells (PBMCs) from patients with ARDS and healthy controls were isolated using Ficoll-Paque density gradient centrifugation.

### Gene set enrichment analysis (GSEA)

GSEA software programs (http://software.broadinstitute.org/gsea/msigdb/index.jsp) were used to determine the enrichment of specific gene sets that from the MSigDB database positively correlated with LPS stimulation in AMs from GSE4088 dataset.

### Establishment of mouse ALI experimental model

WT C57BL/6 mice were randomly instilled intratracheally with PBS (*n* = 12) or LPS (5 mg/kg, *n* = 12) as described previously [42]. After 6 h of LPS or PBS instillation, 6 mice from each group injected intraperitoneally with the SPHK2 inhibitor, ABC294640 (20 mg/kg, dissolved in DMSO); 6 mice from each group received the same volume of DMSO (1 μL in 100 μL). In short, a total of 24 C57BL/6 mice were randomly assigned into 4 groups: PBS group (PBS + DMSO), ABC294640 group (PBS + ABC294640), LPS group (LPS + DMSO) and LPS + ABC294640 group. All mice were sacrificed after 24 h of LPS administration. Bronchoalveolar lavage fluid (BALF) and lung tissue were immediately collected from each animal for the following experiments. The researchers were blinded to the group allocation during the experiment. The procedure of this study was approved by the Animal Care Committee of Zhongshan Hospital, Fudan University and was conducted according to the guidelines for the ethical use of laboratory animals.

### Cell treatments and transfections

The RAW264.7 cells were cultured in Dulbecco’s Modified Eagle Medium (DMEM, Gibco) supplemented with 10% fetal bovine serum (FBS; Gibco) in a humidified 5% CO_2_ incubator at 37 °C. To establish an in vitro ALI model, RAW264.7 cells were stimulated with 1 μg/mL LPS (diluted by PBS) for 6 h, 12 h, and 24 h. To explore the correlation between SPHK2 activity and NLRP3 expression, RAW264.7 cells were pretreated with 0 µM, 5 µM, 10 µM and 50 µM SPHK2 inhibitor (ABC294640) for 30 min, and then stimulated with 1 μg/mL LPS for 24 h. Cell lysates were collected and stored at −80 °C until western blot analysis. For transient transfection, sh-SPHK2 and its corresponding negative control (NC) were diluted in Opti-MEM® medium (Thermo Scientific) and transfected using Lipofectamine 3000 (Invitrogen) according to the manufacturer’s instructions. Cells were then cultured in DMEM medium with 10% FBS after transfection of 6 h.

In a 5% CO_2_ incubator at 37 °C, THP-1 cells were cultured in RPMI-1640 medium (DMEM, Gibco) containing 10% FBS, 1 mM sodium pyruvate, 10 mM Hepes, 25 mM glucose, 100 U/mL penicillin, 100 μg/mL streptomycin and 0.05 mM 2-mercaptoethanol, as well as maintained at a cell density between 0.2 × 10^6^ and 1 × 10^6^ cells/mL. To test the effect of SPHK2 inhibitor (ABC294640) on human monocytes, cells were pretreated with or without 50 µM ABC294640 for 30 min, and then stimulated with 1 μg/mL LPS for 24 h.

### Evaluation of BALF

BALF samples were obtained by washing the lung 3 times with 1 mL 1× PBS via a tracheal cannula (>80% recovery rate). The cells in Lavage samples were pelleted at 3000 rpm for 10 min at 4 °C. The sedimented cells were resuspended, and the total cell count was determined by a haemocytometer. The supernatant was collected for total protein level analysis using a BCA protein assay kit (Beyotime, China).

### Measurement of SOD, MPO and MDA in lung tissues

The frozen lung tissues (right) stored at −80 °C was washed with cold saline, homogenized in lysis buffer and centrifuged at 10,000 × *g* for 10 min at 4 °C. The supernatants were collected to detect the content of superoxide dismutase (SOD), myeloperoxidase (MPO) and malondialdehyde (MDA) by commercial kits (Beyotime, China) following the manufacturer’s protocols.

### Histopathologic evaluation

The left lungs were fixed in 10% formalin and then embedded in paraffin. Some sections (5 μm thick) were stained with hematoxylinnand eosin (H&E) for histopathologic analysis. The images were captured by a light microscope (Olympus IX73, Tokyo, Japan).

### Immunocytochemical staining

After appropriate treatment, the paraffin-embedded mouse lung tissue sections (5 μm thick, left) were incubated with anti-NLRP3 antibody at 4 °C overnight. Subsequently, the sections were washed with PBS and stained with HRP-coupled secondary antibody (CST) at room temperature for 1 h. Slides were then stained with 3,3′-diaminobenzidine (DAB) solution and hematoxylin. The immunohistochemical images were obtained using a light microscope (Olympus IX73, Tokyo, Japan).

### Immunofluorescent staining

After deparaffinization, the 5 μm thickness lung tissue (left) sections were permeabilized with 0.1% Triton X-100 and blocked with 5% bovine serum albumin (BSA) for 30 min. The sections were then incubated with F4/80 (1:200) and CD206 (1:200) antibodies overnight at 4 °C. After washing, the sections were incubated with secondary antibodies for 1 h at room temperature. Cell nuclei were counter-stained with 4’,6- diamidino-2-phenylindole (DAPI).

RAW264.7 cells or THP-1 cells were seeded on a cover glass and treated as indicated. After fixing with 4% paraformaldehyde at room temperature for 15–20 min, the cells were permeabilized with 0.1% Triton X-100 in PBS for another 10 min, and blocked in 5% BSA for 30 min. The coverslips were incubated with CD206 (1:500), iNOS (1:500), Caspase-1 p20 (1:100), NLRP3 (1:500), SPHK2 (1:200), Acetylated-Lysine (1:500) or p53 (1:500) overnight at 4 °C. Thereafter, cells were incubated in secondary antibody and nuclei were stained with DAPI. The images were obtained using a microscope (Olympus IX73, Japan) or a confocal microscope (Fluoview 1000, Olympus, Tokyo, Japan).

### Isolation of nuclear fraction from macrophages

Nuclear fraction of RAW246.7 cells was isolated by nuclear and cytoplasmic extraction kit (Beyotime, Shanghai, China) following the manufacturer’s protocols. After the completing the extraction of nucleoprotein, 5× SDS loading buffer was added (1:4) to samples, which were then boiled at 100 °C for 10 min and stored at 80 °C until western blot analysis.

### HDAC activity assay

Nuclear protein of RAW246.7 cells was extracted with nuclear and cytoplasmic extraction kit (Beyotime, Shanghai, China) according to the manufacturer’s instructions, and HDAC activities upon LPS treatment were evaluated by Histone Deacetylase Assay Kit, Fluorometric (Abnova, USA). Briefly, a total of 10–50 μg of nuclear extract was added to each well, then diluted with ddH_2_O until the final volume was 85 μL (for background reading, add 85 μL ddH_2_O only), or added 2 μL of Trichostatin A into diluted sample for negative control. Besides, 2 μL of HeLa nuclear extract was diluted with 83 μL ddH_2_O for positive control. After incubating with HDAC Fluorometric substrate at 37 °C for 30 min, 10 μL of Lysine Developer was added to stop the reaction. The plate was incubated at room temperature for 30 min, the fluorescence intensity of the wells was measured on a fluorometric plate reader with excitation set at 350–380 nm and emission detection set at 440–460 nm.

### Quantitation of sphingolipids by LC-MS/MS

Sphingolipids were measured by Liquid chromatography tandem-mass spectrometry (LC-MS/MS, 4000 QTRAP, AB Sciex, Framingham, MA, USA) as described previously [10, 12]. Briefly, nuclei were isolated from the RAW264.7 cells as described above, and then washed extensively with PBS. Internal standards were added (0.5nmol each, Sphingolipid Mixture II/LM-6005, Avanti Polar Lipids), lipids extracted, and sphingolipids quantified by LC-MS/MS.

### Determination of mitochondrial reactive oxygen species (mtROS) levels

mtROS levels in PBMCs, RAW264.7 and THP-1 cells were was detected by MitoSOX™ Red superoxide indicator according to the manufacturer’s instructions. Briefly, PBMCs, RAW264.7 or THP-1 cells were loaded with 5 μM MitoSOX Red for 10 min at 37 °C and washed twice with PBS. mtROS levels were determined by measuring the absorbance value at 510/580 nm under a microplate reader. Or, red fluorescence intensity (mtROS; mean fluorescence intensity, MFI) was quantified through FACSCalibur (BD Biosciences).

### Mitochondrial membrane potential (MMP)

MMP was measured using a JC-1 mitochondrial membrane potential assay kit according to the manufacturer’s instructions. RAW264.7 or THP-1 cells (1 × 10^5^) were incubated with JC-1 (10 mg/mL) staining buffer for 20 min at 37 °C and then washed with PBS. Quantification of fluorescence intensity was conducted by FACSCalibur and analyzed using FlowJo software. The ratio of MFI for aggregates (red fluorescence, healthy mitochondria) to monomers (green fluorescence, depolarized mitochondria), or the relative MFI of green fluorescence (for THP-1 cells) was regarded as a marker of MMP loss.

### Enzyme-linked immunosorbent assay (ELISA)

The peripheral blood from patients with ARDS (*n* = 12) and healthy donors (*n* = 3) was centrifuged at 1500 × *g* for 10 min at 4 °C. The serum was collected and stored at −80 °C until use. The cell-free supernatants of RAW264.7 or THP-1 cells were centrifuged at 400 × *g* for 5 min at 4 °C, and then were stored at −80 °C for further analysis. The levels of cleaved caspase-1 (p20) were evaluated by ELISA assay in either serum or cell supernatants according to the manufacturer’s instructions.

### Western blot analysis

BCA Assay was used to measure protein concentration. 30 mg proteins from lung tissues (right), THP-1 cells, RAW264.7 cells and nuclear fraction were separated in 10% or 12.5% SDS-polyacrylamide gel. The polyvinylidene difluoride (PVDF) membranes were blocked with 5% BSA/Tris-buffered saline with Tween-20 (TBST) for 1 h at 37 °C, and the incubated overnight at 4 °C with primary antibodies at the following dilutions: SPHK2 (1:500), p-SPHK2 (1:1000), NLRP3 (1:1000), Caspase-1 (1:200), ASC (1:1000), Caspase-1 p20 (1:200), p53 (1:1000), Acetylated-Lysine (1:1000), acetyl-p53 (1:1000), Histone H3 (1:2000) and GAPDH (1:500). After washing three times with TBST, bands were incubation with appropriate secondary antibodies (1:5000). The bands were detected by ECL (Amersham Pharmacia Biotech, Piscataway, NJ) and quantified using Image J gel analysis software. GAPDH or Histone H3 was used as a loading control. Then, the relative protein levels in each group were normalized against the control group.

### Quantitative reverse transcriptase-PCR (qRT-PCR)

Total RNA was extracted from lung tissues (right) of mice and RAW264.7 cell lysates using TRIzol reagent (Invitrogen, Carlsbad, CA). qRT-PCR was performed on a real-time PCR system (Applied Biosystems 7500HT; Applied Biosystems, Foster City, CA) using SYBR-Green Master Mix Plus (Toyobo, Osaka, Japan) according to the manufacturer’s protocols. The relative expressions level of interleukin-1β (IL-1β), TNF (tumor necrosis factor)-α, inducible nitric oxide synthase (iNOS), cyclooxygenase 2 (COX-2) and interleukin-6 (IL-6) mRNA were normalized by evaluating the GAPDH mRNA level. The specific primers used for the amplification were purchased from Sangon Biotech (Shanghai, China) and are listed in the supplementary material, Table S1.

### Co-immunoprecipitation (Co-IP)

The RAW264.7 cell lysates were immunoprecipitated with 1 μg of anti-p53 antibody overnight at 4 °C, and 30 μL protein A agarose beads (Cell Signaling Technology) was added to the mixture and incubated at 4 °C for 4 h. The agarose beads were collected by centrifugation at 12,000 × *g* for 60 s at 4 °C and washed 3 times with a lysis buffer. The acetylation level of the p53 was analyzed with western blotting using anti-acetylated-lysine antibody. Immunoprecipitation with mouse IgG was taken as negative control.

### Chromatin immunoprecipitation (ChIP) assay

Chromatin was prepared from RAW264.7 cell lysates after formaldehyde-induced cross-linking of endogenous proteins to DNA and shearing. Immunoprecipitation was overnight at 4 °C with sonicated chromatin using anti-p53 monoclonal antibody or control IgG in the presence of G agarose beads (Cell Signaling Technology). DNA was analyzed by qPCR. ChIP primers were designed based on the five predicted p53 binding sites and are listed in the supplementary material, Table S2.

### Luciferase reporter assay

For dual-luciferase reporter assay, 293T cells were co-transfected with indicated plasmids overexpressed p53 or p300 (or negative control), and the pmirGLO luciferase reporter vectors containing wild-type (WT) NLRP3 promoter fragment or fragment covering p53 mutant binding sites (MUT-NLRP3 promoter luciferase reporter). After 48 h of transfection, the luciferase activities of firefly and Renilla were examined the Dual-Luciferase Reporter Assay System (Promega).

### Statistics

Statistical analysis was performed using SPSS 25.0 software (IBM Corporation, USA) and GraphPad Prism 7.0 (GraphPad, USA). Differences between two groups were analyzed by two-tailed *t*-test. Multiple comparisons were performed by one-way analysis of variance (ANOVA) followed by Tukey’s post hoc test. Qualitative data were presented as the means ± standard errors (SEM) from at least three independent experiments. *p* < 0.05 was considered statistically significant.

## Supplementary information

Table S1

Table S2

Figure S1

Figure S2

Figure S3

Original Data File

## Data Availability

All data that support the findings in this study are available from the corresponding author upon reasonable request.
