# Supplementary material for: Nuclear SPHK2/S1P induces oxidative stress and NLRP3 inflammasome activation via promoting p53 acetylation in lipopolysaccharide-induced acute lung injury
Source: Cell Death Discov. 2023 Jan 18;9:12. doi: 10.1038/s41420-023-01320-5 (PMC9847446; doi:10.1038/s41420-023-01320-5)
Supplement: Supplementary file 1 — Table S1 [file 41420_2023_1320_MOESM1_ESM.docx]

**Table S1**

Primer Sequences for Real-Time Quantitative RT-PCR

| **Primer name** | **Sequence** |
| --- | --- |
| TNF-α-Forward (mouse) | CTCCTGGTATGAGATAGC |
| TNF-α-Reverse (mouse) | GTTGTACCTTGTCTACTCCC |
| iNOS-Forward (mouse) | AACATCAGGTCGGCCATCAC |
| iNOS-Reverse (mouse) | AGCCTAGGTCGATGCACAAC |
| COX-2-Forward (mouse) | AACCGAGTCGTTCTGCCAAT |
| COX-2-Reverse (mouse) | CTAGGGAGGGGACTGCTCAT |
| IL-6-Forward (mouse) | GTGGCTAAGGACCAAGACCA |
| IL-6-Reverse (mouse) | GGTTTGCCGAGTAGACCTCA |
| IL-1β-Forward (mouse) | GGGCCTCAAAGGAAAGAATC |
| IL-1β-Reverse (mouse) | TACCAGTTGGGGAACTCTGC |
| GADPH- Forward (mouse)  GADPH- Reverse (mouse) | ACCAGGTGGTCTCCTCTGAC  TGCTGTAGCCAAATTCGTTG |
| GADPH- Forward (human) | GAGTCAACGGATTTGGTCGT |
| GADPH- Reverse (human) | TGGGTGGAATCATATTGGAA |
| NLRP3- Forward (human) | GATCTTCGCTGCGATCAACA |
| NLRP3- Reverse (human) | GGGATTCGAAACACGTGCATTA |
| SPHK2- Forward (human) | CCAGTGTTGGAGAGCTGAAGGT |
| SPHK2- Reverse (human) | GTCCATTCATCTGCTGGTCCTC |
