# Supplementary material for: Nuclear SPHK2/S1P induces oxidative stress and NLRP3 inflammasome activation via promoting p53 acetylation in lipopolysaccharide-induced acute lung injury
Source: Cell Death Discov. 2023 Jan 18;9:12. doi: 10.1038/s41420-023-01320-5 (PMC9847446; doi:10.1038/s41420-023-01320-5)
Supplement: Supplementary file 2 — Table S2 [file 41420_2023_1320_MOESM2_ESM.docx]

**Table S2**

Specific PCR primer sequences for predicting binding site sequences

| Primer | Sequence（5’-3’） | | Tm | CG% | Product |
| --- | --- | --- | --- | --- | --- |
| Target 1 | sense | GTTCACAATGTTTCCCAAGCC | 59.4 | 47.6 | 110 |
|  | antisense | GTACACTACCACGCCTGGCC | 60.2 | 65 |  |
| Target 2 | sense | GCCAGGGCTACAAAGAGAGG | 59 | 60 | 216 |
|  | antisense | GTCCAATCTCTGAAACCCGC | 59.3 | 55 |  |
| Target 3 | sense | GCTTCTGTCCCTGAATCAGCA | 1280 | 60.1 | 166 |
|  | antisense | CTGCTTCTCTGCCACCCTTG | 1445 | 60.6 |  |
| Target 4 | sense | ACAGGAGATGGAGAACACTTGG | 58.1 | 50 | 208 |
|  | antisense | GGAAAAAGTGAAACCTCAGGG | 58.1 | 47.6 |  |
| Target 5 | sense | ACTGCCAATCCGTCTTTGACA | 60.4 | 47.6 | 126 |
|  | antisense | AGCCTTGGGTACAAATAAAGGG | 59.7 | 45.5 |  |
