# Supplementary material for: Nuclear SPHK2/S1P induces oxidative stress and NLRP3 inflammasome activation via promoting p53 acetylation in lipopolysaccharide-induced acute lung injury
Source: Cell Death Discov. 2023 Jan 18;9:12. doi: 10.1038/s41420-023-01320-5 (PMC9847446; doi:10.1038/s41420-023-01320-5)
Supplement: Supplementary file 3 — Figure S1 [file 41420_2023_1320_MOESM3_ESM.docx]

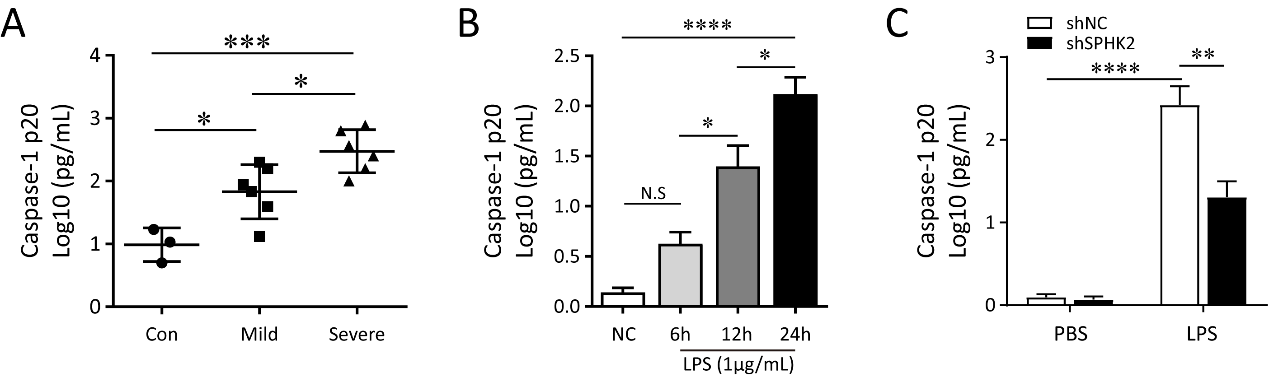


**Fig. S1**

**Determination of caspase-1 p20 levels through ELISA**

(A) Increased caspase-1 p20 protein concentrations in serum of ARDS patients compared with those of the heathy donors; (B) RAW264.7 cells were stimulated with 1μg/mL LPS for 0h (NC), 6h, 12h and 24h. The levels of caspase-1 p20 in the supernatant were measured; (C) shSPHK2 or shNC transfected RAW264.7 cells were stimulated with 1μg/mL LPS for 24h, and the levels of caspase-1 p20 in the supernatant were detected. Data were presented as the means ± SEM from at least 3 independent experiments. *p<0.05; **p<0.01; ***p<0.005; ****p<0.001; N.S, not significant.
