# Supplementary material for: Nuclear SPHK2/S1P induces oxidative stress and NLRP3 inflammasome activation via promoting p53 acetylation in lipopolysaccharide-induced acute lung injury
Source: Cell Death Discov. 2023 Jan 18;9:12. doi: 10.1038/s41420-023-01320-5 (PMC9847446; doi:10.1038/s41420-023-01320-5)
Supplement: Supplementary file 4 — Figure S2 [file 41420_2023_1320_MOESM4_ESM.docx]

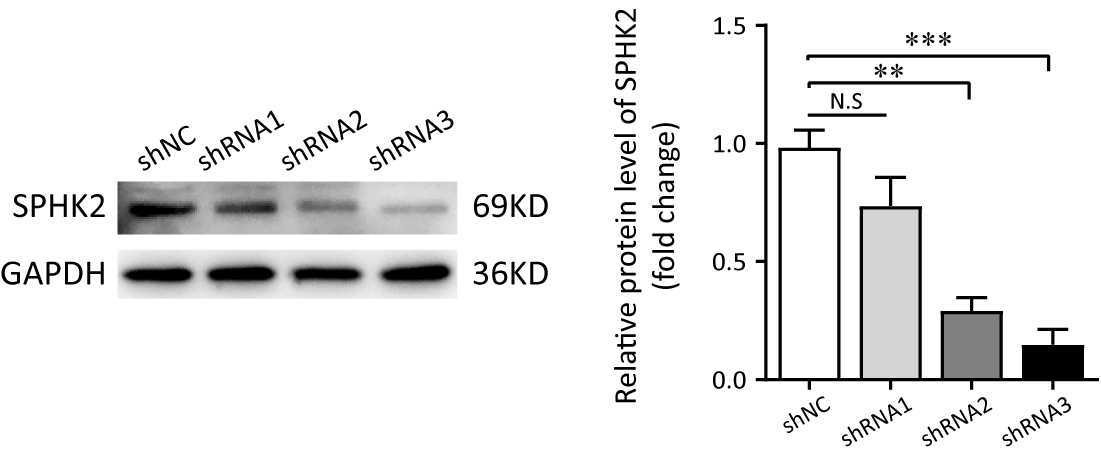


**Fig. S2**

**Knockdown efficiency of shRNAs in RAW264.7 cell were detected via immunoblotting**

The levels of SPHK2 protein in RAW264.7 cells were determined by western blot. Macrophages were transfected with SPHK2 shRNA1~3 until 48h before the next experimental step.
