# Supplementary material for: Nuclear SPHK2/S1P induces oxidative stress and NLRP3 inflammasome activation via promoting p53 acetylation in lipopolysaccharide-induced acute lung injury
Source: Cell Death Discov. 2023 Jan 18;9:12. doi: 10.1038/s41420-023-01320-5 (PMC9847446; doi:10.1038/s41420-023-01320-5)
Supplement: Supplementary file 5 — Figure S3 [file 41420_2023_1320_MOESM5_ESM.docx]

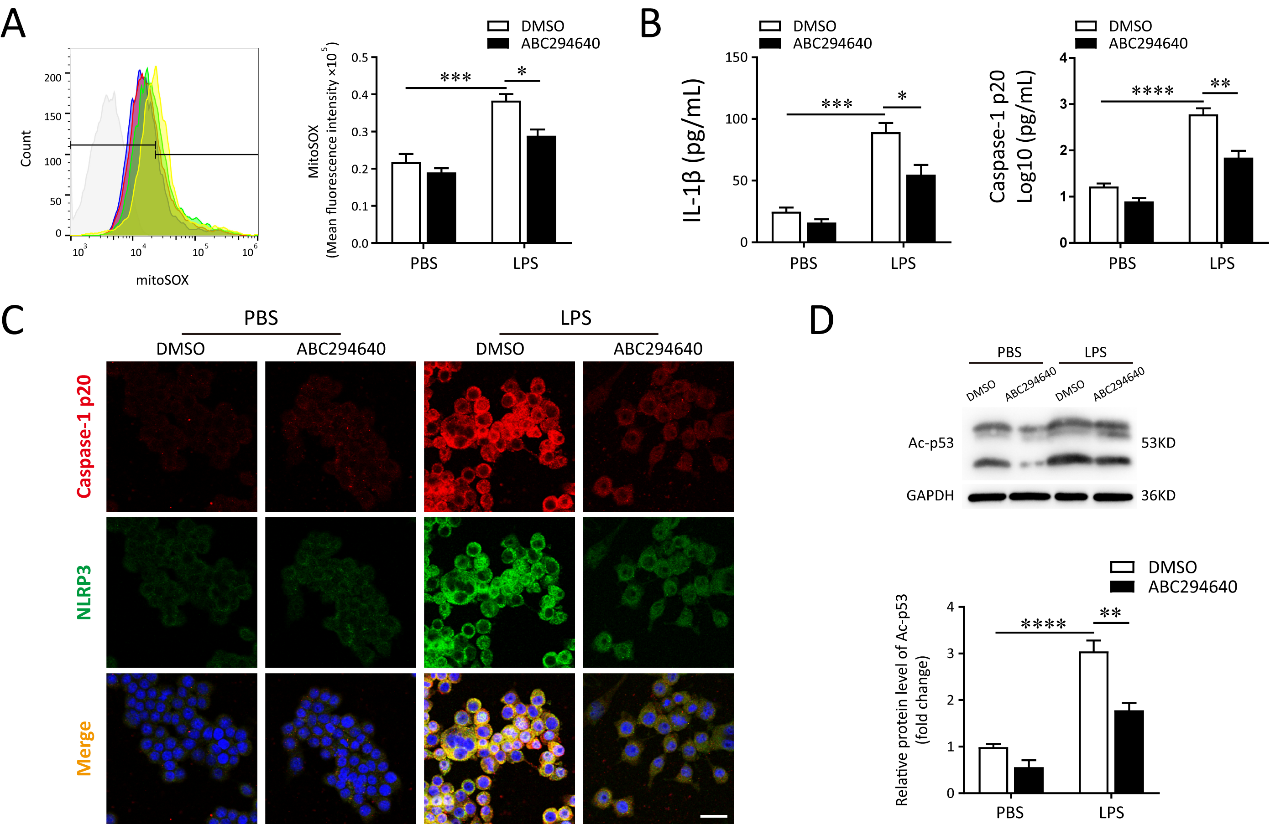


**Fig. S3**

**Inhibition of SPHK2 decreases LPS-triggered oxidative stress, NLRP3 inflammasome activation and p53 acetylation in THP-1**

(A) THP-1 cells were stained with MitoSOX and analyzed by flow cytometry. LPS-induced mtROS production was decreased by SPHK2 inhibition. (B) Levels of IL-1β and caspase-1 p20 in the supernatant were determined by ELISA. (C) Representative confocal microscopic images of THP-1 cells co-localization with Caspase-1 p20 (red) and NLRP3 (green). bar = 25μm. (D) Cell lysates were immunoblotted for ac-p53 proteins in THP-1 cells. Data were presented as the means ± SEM from 3 independent experiments. *p<0.05; **p<0.01; ***p<0.005; ****p<0.001.
