# Supplementary material for: Nuclear SPHK2/S1P induces oxidative stress and NLRP3 inflammasome activation via promoting p53 acetylation in lipopolysaccharide-induced acute lung injury
Source: Cell Death Discov. 2023 Jan 18;9:12. doi: 10.1038/s41420-023-01320-5 (PMC9847446; doi:10.1038/s41420-023-01320-5)
Supplement: Supplementary file 6 — Original Data File [file 41420_2023_1320_MOESM6_ESM.docx]

Figure 1


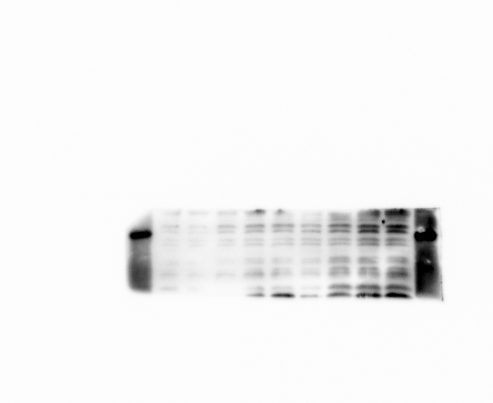
ASC



CASP1


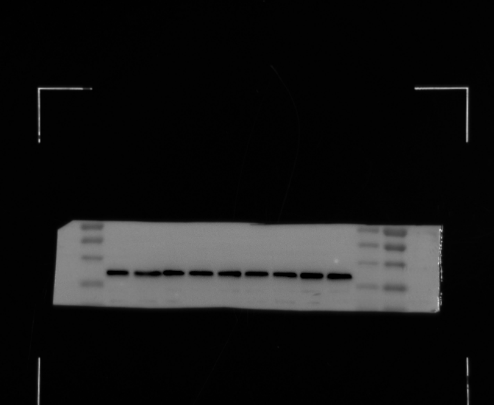
GAPDH


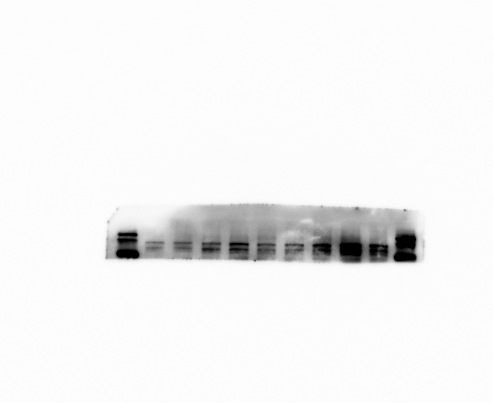
NLRP3


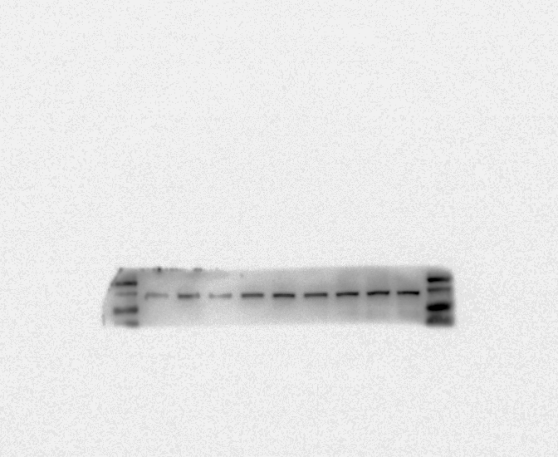
SPHK2

Figure 2A


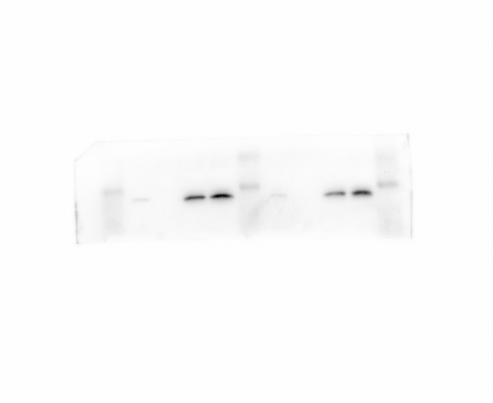
ASC


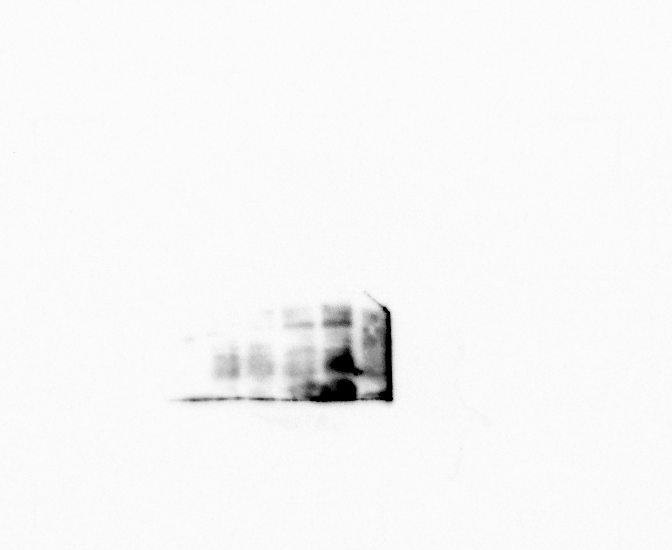
CASP1 P20


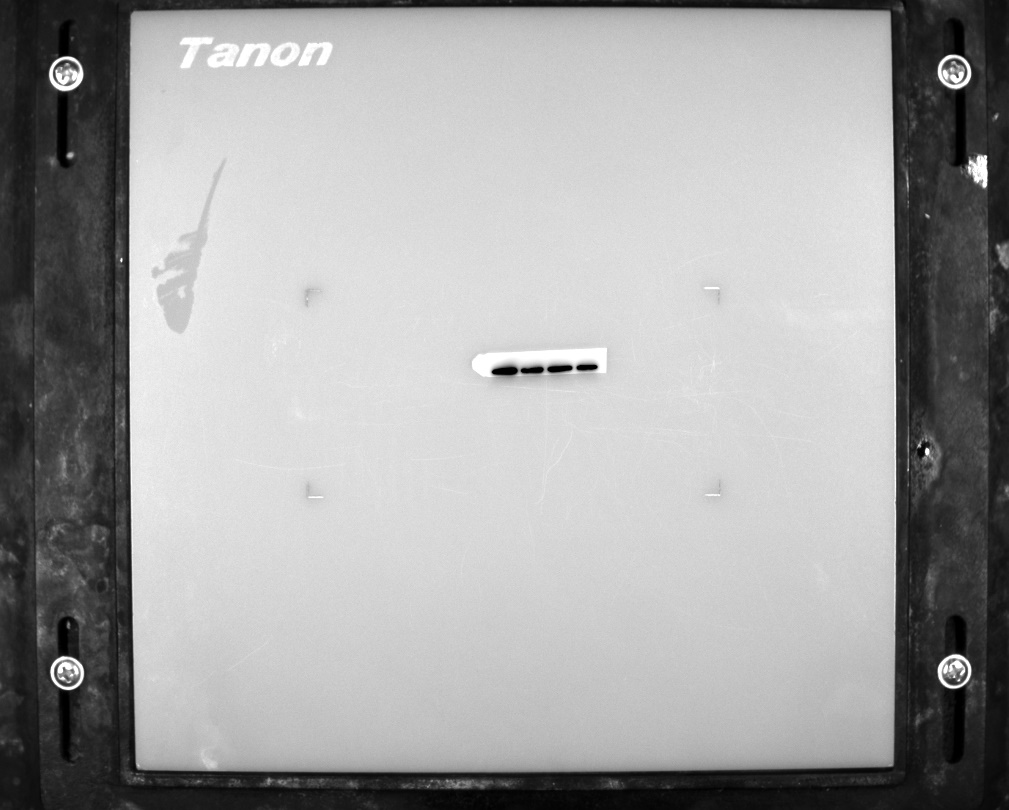
GAPDH


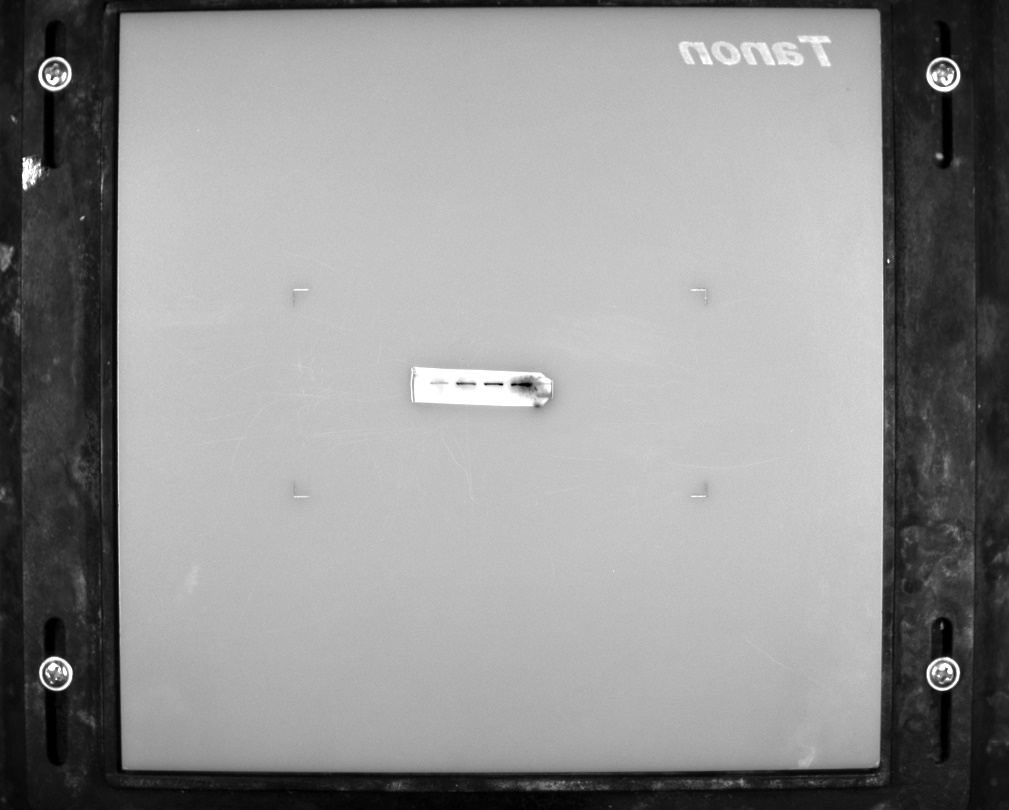
NLRP3

Figure 2B


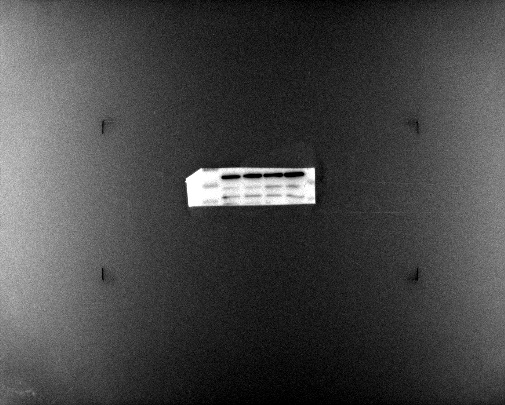
GAPDH


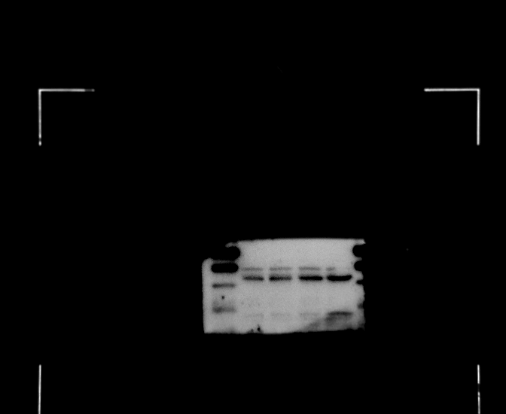
SPHK2

Figure 2C


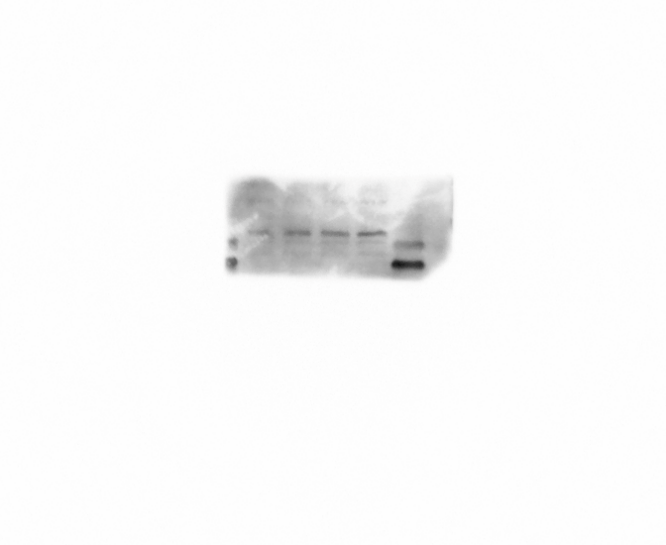
p-SPHK2


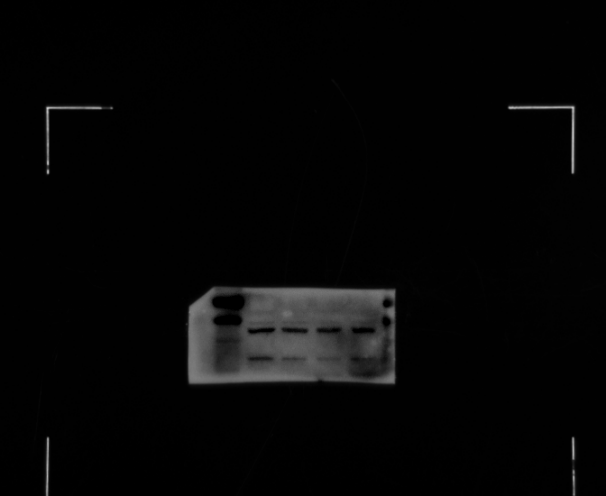
SPHK2

Figure 3D


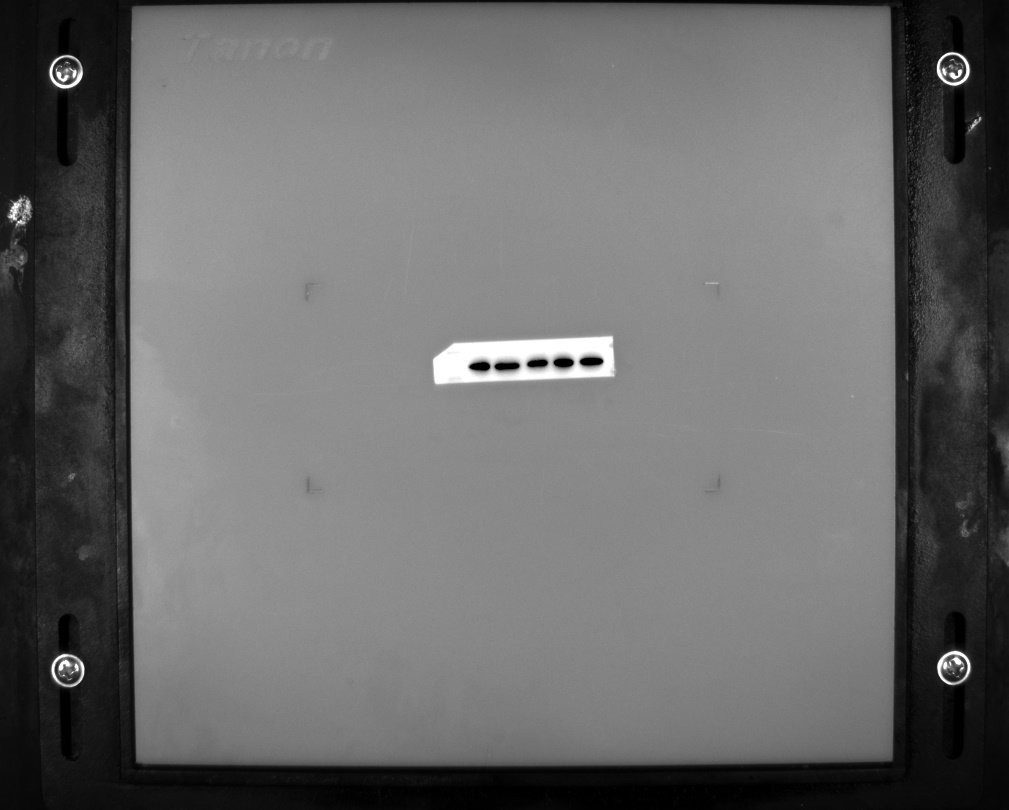
GAPDH


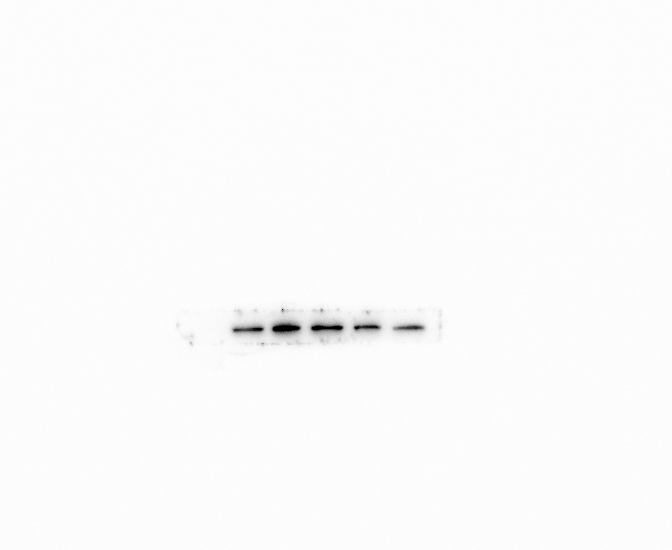
NLRP3

Figure 3E


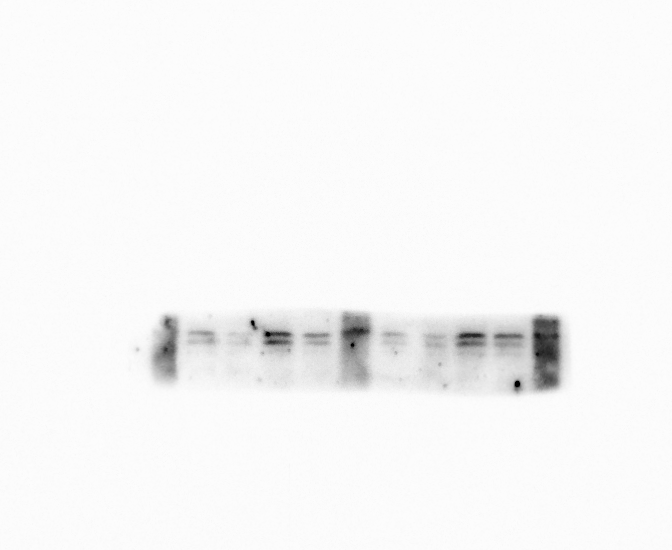
ASC


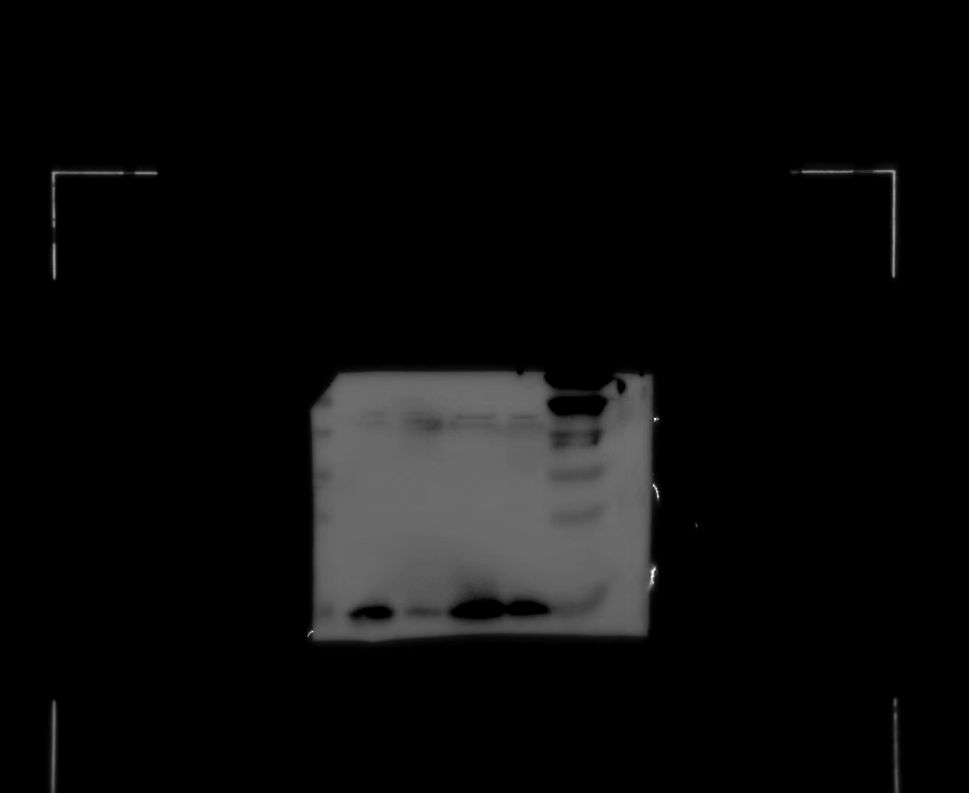
CASP1


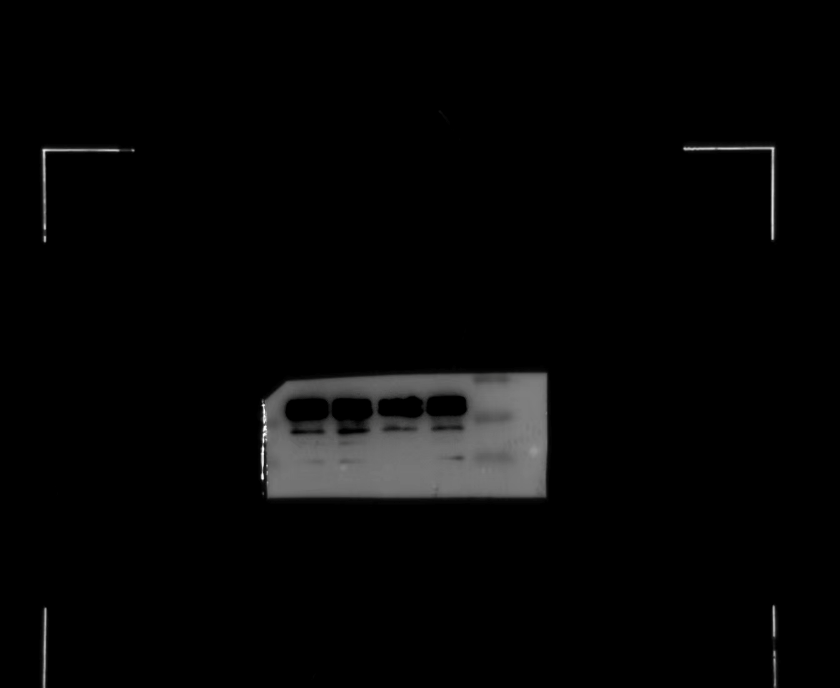
GAPDH


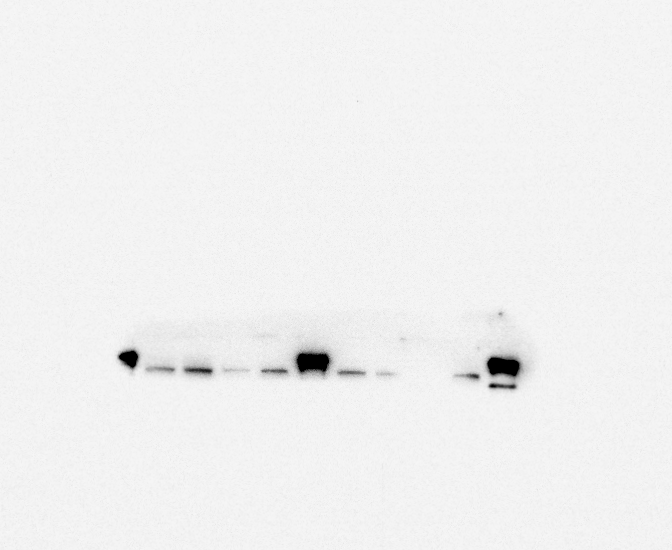
NLRP3

Figure 4B


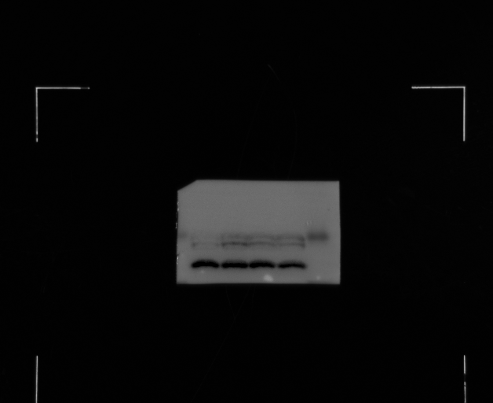
H3


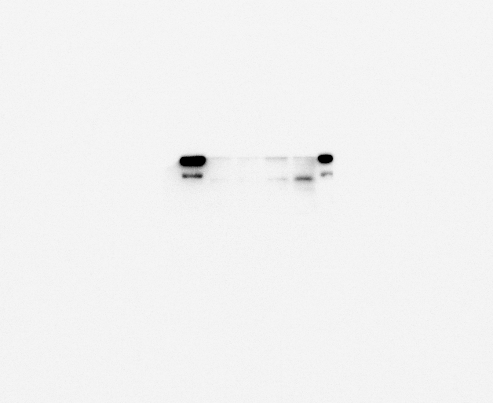
P53


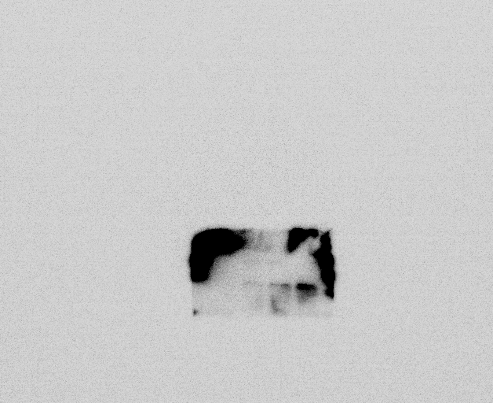
SPHK2

Figure 4G


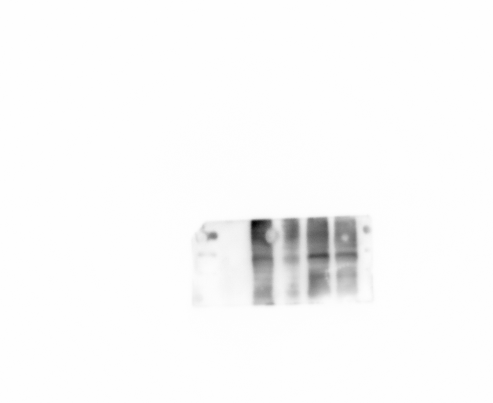
Ac


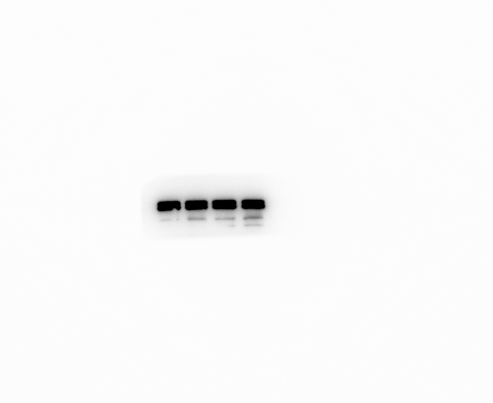
GAPDH


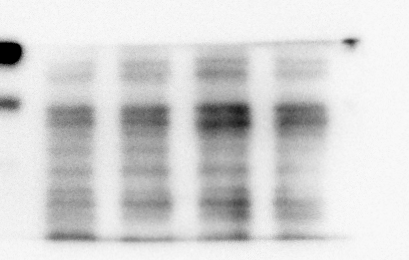
P53


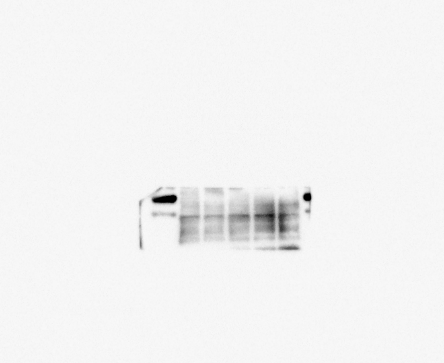
P53

Figure 8A


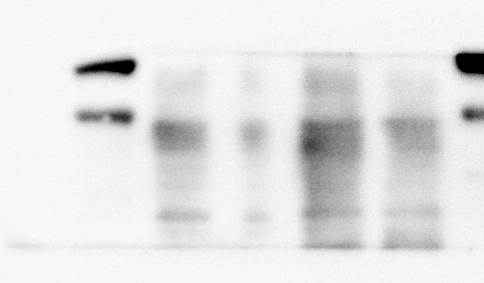
Ac-p53


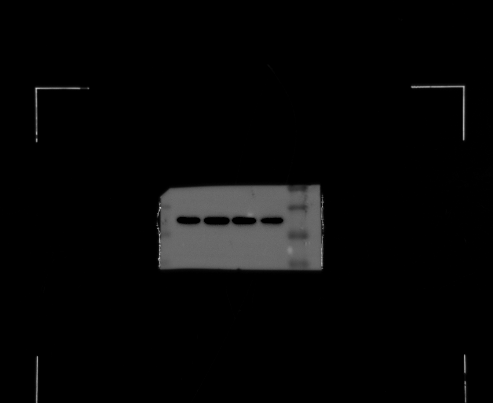
GAPDH

Figure 8D


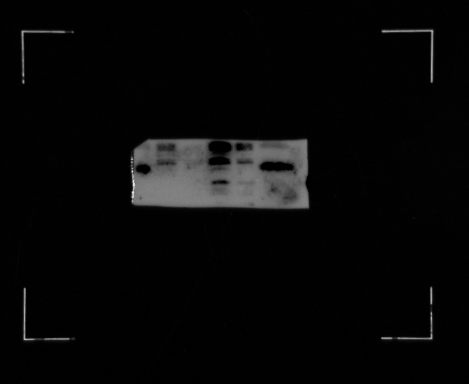
ASC


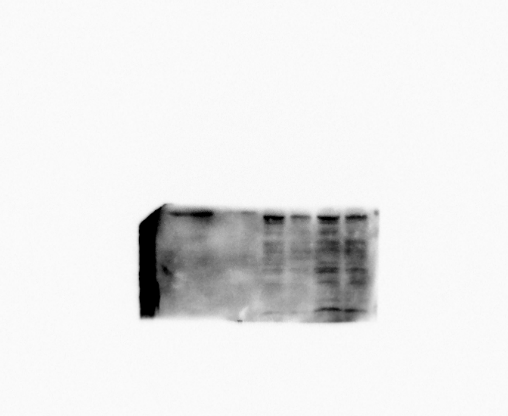
CASP1


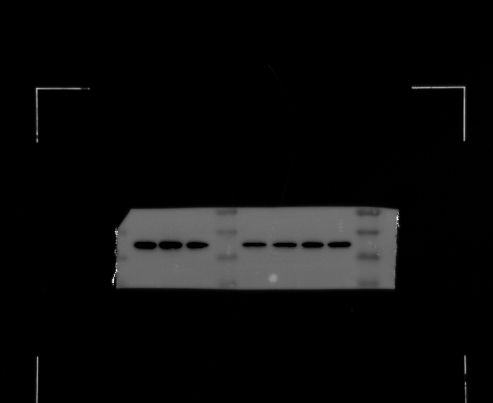
GAPDH


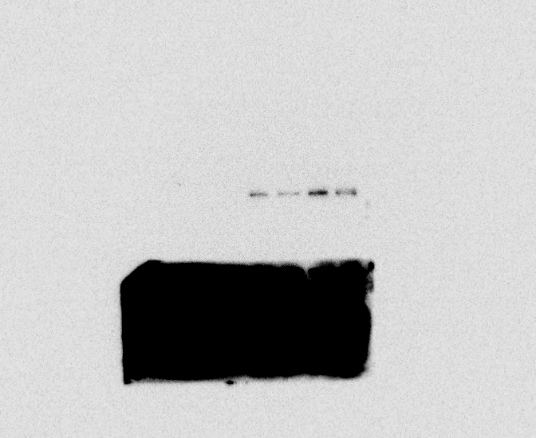
NLRP3

Figure S2


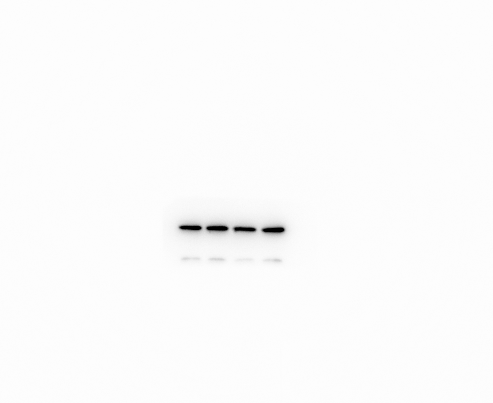
GAPDH


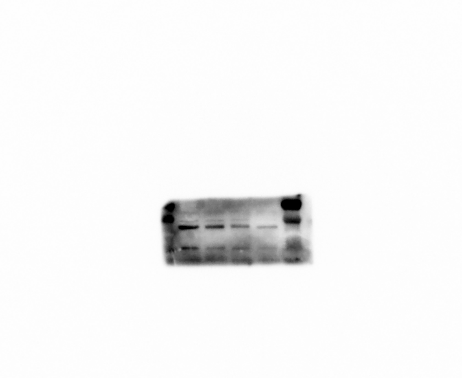
SPHK2

Figure S3


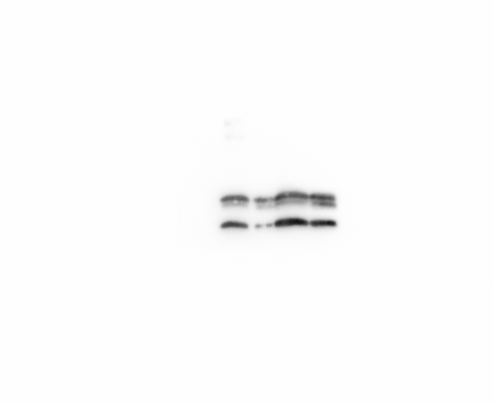
Ac-P53



GAPDH
